# A novel transfer learning framework for sorghum biomass prediction using UAV-based remote sensing data and genetic markers

**DOI:** 10.3389/fpls.2023.1138479

**Published:** 2023-04-11

**Authors:** Taojun Wang, Melba M. Crawford, Mitchell R. Tuinstra

**Affiliations:** ^1^ School of Electrical and Computer Engineering, Purdue University, West Lafayette, IN, United States; ^2^ Lyles School of Civil Engineering, Purdue University, West Lafayette, IN, United States; ^3^ Department of Agronomy, Purdue University, West Lafayette, IN, United States

**Keywords:** hyperspectral, LiDAR, biomass prediction, genetic markers, long short-term memory, recurrent neural network, transfer learning

## Abstract

Yield for biofuel crops is measured in terms of biomass, so measurements throughout the growing season are crucial in breeding programs, yet traditionally time- and labor-consuming since they involve destructive sampling. Modern remote sensing platforms, such as unmanned aerial vehicles (UAVs), can carry multiple sensors and collect numerous phenotypic traits with efficient, non-invasive field surveys. However, modeling the complex relationships between the observed phenotypic traits and biomass remains a challenging task, as the ground reference data are very limited for each genotype in the breeding experiment. In this study, a Long Short-Term Memory (LSTM) based Recurrent Neural Network (RNN) model is proposed for sorghum biomass prediction. The architecture is designed to exploit the time series remote sensing and weather data, as well as static genotypic information. As a large number of features have been derived from the remote sensing data, feature importance analysis is conducted to identify and remove redundant features. A strategy to extract representative information from high-dimensional genetic markers is proposed. To enhance generalization and minimize the need for ground reference data, transfer learning strategies are proposed for selecting the most informative training samples from the target domain. Consequently, a pre-trained model can be refined with limited training samples. Field experiments were conducted over a sorghum breeding trial planted in multiple years with more than 600 testcross hybrids. The results show that the proposed LSTM-based RNN model can achieve high accuracies for single year prediction. Further, with the proposed transfer learning strategies, a pre-trained model can be refined with limited training samples from the target domain and predict biomass with an accuracy comparable to that from a trained-from-scratch model for both multiple experiments within a given year and across multiple years.

## Introduction

Population growth and the increasing consumption of energy in a world economy that seeks to reduce dependence of fossil fuels have incentivized development of biofuels as an environmentally friendly, renewable energy source that can help fulfill the global demand (Rodionova et al., 2017). Sorghum, a C4 plant, can efficiently use water and nutrients to produce biomass-based fuels with lower CO_2_ emissions than fossil fuels (van der Weijde et al., 2013). With recent developments in modern biotechnology, it is possible to create numerous genotypes of sorghum and select those that maximize biomass production. However, biomass is traditionally measured by destructive sampling, which is extremely time- and labor-consuming, and cannot provide a timely evaluation of this complex phenotypic trait, especially in large breeding programs. Recent advances in remote sensing (RS) technology have enabled field-based high-throughput phenotyping. Platforms like unmanned aerial vehicles (UAVs) and ground-based systems can carry multiple sensors, including RGB cameras, multispectral, hyperspectral, thermal infrared scanners, and light detection and ranging (LiDAR) units, and maneuver in the field to acquire high-quality data at high spatial resolution (Yang et al., 2017; Xie and Yang, 2020; Feng et al., 2021). These platforms have great potential for precision agriculture applications due to their capability to acquire high spatial and temporal resolution data “on demand” *via* efficient, non-invasive relatively low-cost field surveys. UAV data have been used to estimate many phenotypic traits, including plant height (Pérez-Ruiz et al., 2020), canopy cover (Sun et al., 2018; Masjedi et al., 2020), leaf area index (Ribera et al., 2018; Comba et al., 2020; Nazeri et al., 2021), and biomass (Ballesteros et al., 2018; Masjedi et al., 2019), replacing traditional in-field phenotyping.

Researchers continue to explore approaches to model the relationships between RS data and more complex phenotypes, such as biomass (Lu, 2006; Chao et al., 2019). This is extremely challenging because the relationship between time-series RS data and biomass is complex and is highly variable within and between varieties in a breeding experiment. These interactions also vary throughout the growing season due to environmental conditions, even with consistent management practices. Additionally, the number of ground reference samples for training models is very limited. In the breeding experiments, hundreds of genotypes are planted with limited replicates. Recently, the Long Short-Term Memory (LSTM), a recurrent neural network (RNN) architecture, has been demonstrated to be effective for dealing with continuous input. For agricultural applications, prior studies that adopted this network structure achieved high accuracy in crop yield prediction (Khaki et al., 2020; Gong et al., 2021; Fan et al., 2022) and plot-level biomass prediction (Masjedi et al., 2019). The main limitations of current LSTM-based biomass prediction models are: i) extraction and selection of robust, appropriate features are critical as having highly correlated features could degrade the performance of the prediction model; ii) current models do not incorporate the genetic marker data due to its high dimensionality compared with other features (phenotypic information, environmental conditions, or management practices); iii) a prediction model pre-trained on a single year of RS data does not generalize well on another trial from a different year or location due to differences in genotypes and environmental conditions.

In this study, an LSTM-based RNN model is proposed for plot-level sorghum biomass prediction using RS data (representing phenotypic traits), weather data, and genotypic information. Feature importance is first evaluated to identify and remove the highly correlated features from the prediction model. A strategy for reducing the dimensionality of the genetic marker data and incorporating this information into an LSTM-based RNN prediction model is proposed. In response to the need for generalization and to limit requirements for ground reference data, transfer learning techniques are developed to refine a pre-trained model using limited training samples from a new trial with additional genotypes, different environmental conditions or management practices. The performance of the proposed approaches is evaluated over a sorghum breeding experiment that contained more than 600 testcross hybrids and was planted in multiple years. The remainder of this paper is organized as follows: Section *Related work* provides a review of prediction models using RS data and genotype information, along with different transfer learning strategies; Section *Materials and methods* describes the study sites, datasets, and the methodologies including feature extraction, the prediction model, and transfer learning strategies; The experimental results are presented and discussed in Section *Experimental results and discussion*; Conclusions of the study are summarized in Section *Conclusions and discussion*, which also includes recommendations for future work.

## Related work

### Feature extraction/selection

Compared to data in the two- or three-dimensional physical space, high dimensionality inputs result in “the curse of dimensionality” (Altman and Krzywinski, 2018). The quantity of data required to obtain reliable results grows exponentially with dimensionality due to the sparsity caused by high dimensionality (Zimek et al., 2012). Hyperspectral data with hundreds of bands are not only high dimensional, but many bands are also highly correlated. It is necessary to reduce the number of features due to the limited quantity of training samples; this is achieved either by feature extraction or feature selection techniques. Feature selection focuses on selecting a subset of features that can efficiently represent the original feature space and provide good classification/prediction results. A comprehensive comparison of feature selection criteria is available in Khalid et al. (2014). In contrast, feature extraction aims at projecting the original feature space into a new space with lower dimensionality. Although the transformed features may not directly represent physical characteristics related to the original data, the goal of feature extraction is similar to feature selection: to reduce complexity and represent the original feature space in a simple way. As noted in the survey article (Khalid et al., 2014), the success of feature extraction/selection methods is strongly related to datasets, so no single method is globally preferred.

### Biomass prediction model

Researchers have developed predictive models for biomass using RS data *via* two primary approaches. The first strategy incorporates limited RS data products into crop simulation models, such as the Agricultural Production Systems sIMulator (APSIM) (Hammer et al., 2010). For example, (Yang K. W. et al. (2021) parameterized the APSIM crop growth model with canopy cover estimated from RGB remote sensing data products to predict sorghum biomass; Zheng et al. (2019) proposed an improved APSIM wheat model to predict traits related to canopy development more accurately. Combining crop simulation models with machine learning or deep learning approaches is an active area of research (Messina et al., 2018; Feng et al., 2020; Xie and Huang, 2021; Jeong et al., 2022). Mechanistic models incorporate biological processes, providing opportunities to generalize empirical models that are based on observed conditions, but require extensive field data input for tuning beyond general conditions.

The second approach focuses on the development of empirical models, such as support vector regression (SVR), random forest (RF), and partial least squares regression (PLSR), which depend heavily on training sample size and RS data type (Fassnacht et al., 2014). For example, Huntington et al. (2020) used a variation of RF to predict sorghum yields; Han et al. (2019) and Masjedi et al. (2020) both evaluated multiple classical machine learning algorithms, e.g. SVR, PLSR, RF, and multiple regression, for above-ground biomass prediction. None of the approaches were able to fully model the relationship between time series RS data/environment inputs and biomass. In addition, they do not fully leverage data from different remote sensing modalities or effectively incorporate genetic information, environmental conditions, and management practices, and are not easily generalizable.

Recently, deep learning approaches have been investigated for building complex physical models with RS data (Zhu et al., 2017). Recurrent neural networks contain a rich class of dynamic models that can process variable length sequences of inputs (Graves, 2013). Among them, the LSTM is an RNN architecture that has been demonstrated to be effective for dealing with continuous input such as video (Ullah et al., 2017), speech recognition (Graves et al., 2013), and natural language modeling (Sundermeyer et al., 2012). Due to the special architecture, an adaptive “forget gate” (Gers et al., 2000)enables an LSTM cell to “rest itself “when its contents are “out of date and become useless”. Researchers have integrated both genotype and phenotype related information into the LSTM-based prediction model. For example, Khaki et al. (2020) developed an LSTM-based crop yield prediction model for different environments with a large quantity of historical soil, weather, and management practice data from 1980 to 2018 within 13 states in the United States as training samples. Shook et al. (2021) utilized time-series weather data, clustered genotype information and maturity group to predict field-scale soybean yield over 150 locations. In the context of plant breeding programs, breeders aim to develop the traits of crops to produce desired characteristics (Poehlman, 2013). They seek to focus on specific genotype candidates for which it would typically be impossible to obtain years of historical RS data. Masjedi et al. (2019) proposed an LSTM-based RNN model, which achieved accurate biomass prediction with multi-temporal RS data in a single year. However, their model trained with a single year of RS data could not achieve high accuracy when testing on another trial with different genotypes or environmental conditions. While potentially useful for post-season evaluation under given conditions, the model lacked generalizability.

### Transfer learning

Transfer learning in deep learning seeks to transfer knowledge learned from a source domain to a target domain while using limited training samples from the target domain (Pan and Yang, 2010). Unlike semi-supervised methods, which assume unlabeled and labeled data sets have the same distribution, transfer learning allows the target domain to have different distributions from the source domain. The goal is to efficiently update the model to accommodate these differences. The three general categories of transfer learning approaches are: instance-based, mapping-based, and network-based (Tan et al., 2018). The instance-based strategy assumes that appropriate weights assigned to a partial dataset of source domain data can be added into the training set in the target domain. Thus, the goal of this strategy is to identify data in the source domain similar to the target domain and re-weight these points (Dai et al., 2007; Jiang and Zhai, 2007; Li et al., 2017). The mapping-based strategy assumes both source and target domains can be mapped into a new space, and data from the two domains become more similar in the new data space. Therefore, an important issue of this strategy is to measure the difference in distributions or similarity between domains. The network-based deep learning strategy, which is the most popular approach for artificial neural networks, refers to partially using the pre-trained network from the source domain, and fine-tuning the parameters with training samples from the target domain. The assumption for the network-based strategy is that the front layers of the pre-trained network can be treated as a feature extractor. A common usage of this strategy for RGB image related purposes involves freezing front layers of convolutional neural networks (CNNs) trained on the diverse RGB ImageNet dataset, then randomly initializing the remaining layers and training in target domains with limited training samples. However, the transferability varies with different network structures (Yosinski et al., 2014); AlexNet, VGG, and ResNet all perform well with the network-based deep learning strategy. Researchers have also explored the transferability of time-series prediction models; for example, Zoph et al. (2016) used an LSTM-based RNN trained with high-resource language data and refined it with very little low-resource language data as the target domain. Similarly, Dong et al. (2019) proposed a bi-directional RNN model which was pre-trained with a general Chinese corpus as the feature extractor, then fine-tuned with a Chinese electronic medical record corpus as the target domain to extract more accurate features.

Transfer learning strategies have also been used in agricultural studies (Wang and Su, 2022). Abdalla et al. (2019) evaluated three transfer learning methods using a VGG-based encoder net for oilseed rapes image segmentation; Cai et al. (2020) proposed a modified U-Net architecture with transfer learning strategy for detecting plant locations; (Yang S. et al. (2021) utilized transfer learning with Mask R-CNN for soybean seed segmentation. For the RNN architecture, Wang et al. (2018) proposed a deep transfer learning framework with LSTM-based RNN for soybean yield prediction in different countries with satellite imagery. Later, Kaya et al. (2019) applied a transfer learning strategy to an RNN-CNN combined model for leaf classification.

### Few-shot learning

The concept of supervised learning with a limited number of examples from the target domain is called Few-Shot Learning (FSL) (Wang et al., 2020). Compared to the most current machine learning algorithms, humans can efficiently use prior knowledge and learn new tasks with a few examples. Thus, FSL has been proposed recently as a machine learning paradigm. For instance, FSL in a classification scenario is referred to as “k-shot, N-way”, aiming to train classifiers for N classes with only k training samples per class. The supervised FSL strategies can be categorized based on three perspectives, data, model, and algorithm. The data perspective aims at using prior knowledge to enrich the limited label data in the target domain. Some common strategies for image related tasks include classical augmentation (translation, flipping, scaling, cropping, rotation, etc). Designing a reasonable strategy depends heavily on domain knowledge and might not be applicable to other data sets (Wang et al., 2020). The model perspective simplifies the problem by using prior knowledge and results in a much smaller hypothesis space. Usually, a small hypothesis space cannot represent real-world problems very well, but with the guidance of prior knowledge, the empirical risk can be minimized. Four types of methods have been proposed based on different prior knowledge: multitask learning (Caruana et al., 1997), learning through feature embedding (Jia et al., 2014), learning with external memory (Graves et al., 2014), and generative modeling (Reed et al., 2017). The algorithm perspective searches for the best parameters by refining existing parameters. The existing parameters found by prior knowledge provide a good initialization, and the resultant search steps are guided by prior knowledge. To avoid overfitting, multiple regularization methods such as early-stopping and selectively updating the parameters can be used. The FSL has also been used in many agriculture studies, for example plant counting, pest detection, and plant disease recognition (Karami et al., 2020; Yang et al., 2022).

Nevertheless, generating ground reference data in the target domain is still time- and labor- consuming, especially for tasks like collecting fresh biomass described in this paper. It is important to identify a small number of the most informative training samples in the target domain. In this paper, two transfer learning strategies are proposed for an LSTM-based RNN-G sorghum biomass prediction model that incorporates both time-series and static features extracted from RS data, weather data, and genetic marker data.

## Materials and methods

### Field trials and ground reference data

**Figure 1:**
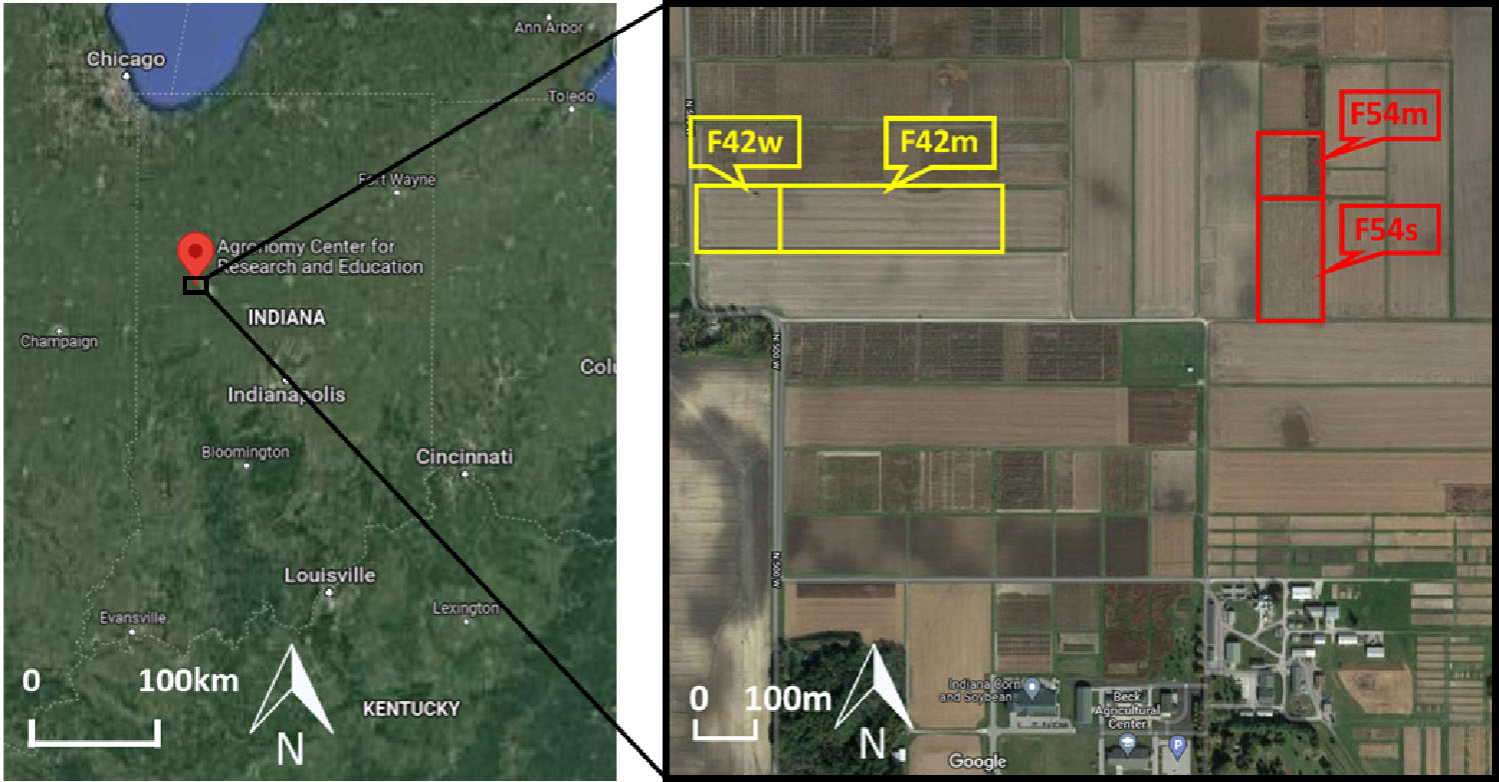
Locations (highlighted by yellow and red boxes) of sorghum experiments at the Purdue University**’**s Agronomy Center for Research and Education (ACRE). *Cal* panels were planted in Fields F42w and F54m, and *Tc* panels were planted in Fields F42m and F54s on alternating years.

The field surveys for this study were conducted over sorghum breeding trials at Purdue University’s Agronomy Center for Research and Education (ACRE) in Indiana, USA (**Figure 1**). The field surveys included the following trials: the sorghum biodiversity testcross (hereafter, Tc) panel in 2018, 2019, and 2020, and the sorghum biodiversity testcross calibration (hereafter, Cal) panel in 2019, 2020, and 2021, as listed in **Table 1**. The Tc panel consisted of 630 hybrid genotypes with two replicates in 4 row plots. The same hybrids were planted over the three years. The Cal panels contained 72 hybrid genotypes with two replicates (in 2019 and 2020) and three replicates (in 2021) in 12 row plots. The 72 hybrids in the Cal panel, which were representative testcrosses selected from the Tc panel, were destructively sampled multiple times during the growing season, providing a time series of ground reference data. Field trials were conducted each year using a randomized complete block design.

**Table 1:** Multi-year field survey information.

Trial	Year	Field	Number of plots	Number of hybrids	Sowing date	Available biomass date
Tc	2018	F54m	1260	630	05/08	08/02
Tc	2019	F42m	1260	630	06/04	09/12
Cal	2019	F42w	144	72	06/04	08/09, 09/05
Tc	2020	F54s	1260	630	05/12	08/19
Cal	2020	F54m	144	72	05/13	07/14, 08/11
Cal	2021	F42w	216	72	05/23	08/03, 09/03

**Figure 2:**
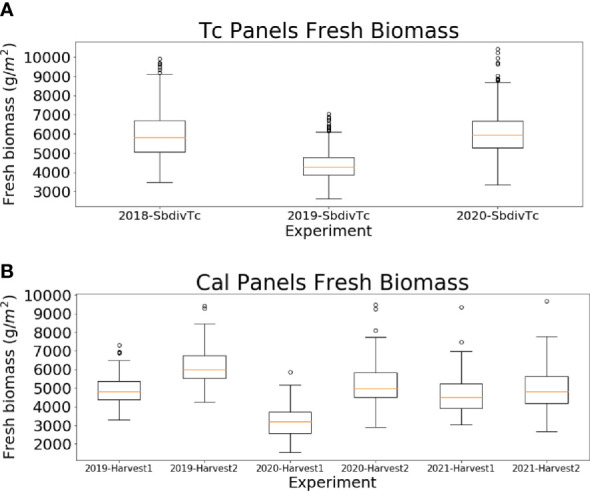
Box plot for **(A)** the end-of-season fresh biomass data for the *Tc* panels in 2018, 2019 and 2020 and **(B)** the two machine harvested fresh biomass data for the *Cal* panels in 2019, 2020 and 2021.

The above-ground biomass data were destructively collected using a Wintersteiger Cibus 2-row Biomass Harvester (Wintersteiger Inc., Salt Lake City, UT, USA). Approximately 500 g of the shredded sorghum plant material in each plot was used to determine fresh weight, dry weight and moisture content. For the Tc panel, rows 2 and 3 of each plot were harvested only once at the end of season. For the Cal panel, biomass data were collected two times by the machine during the growing season at approximately 60-70 (rows 5 and 6) and 90-100 days (rows 2 and 3) after sowing (DAS). The harvest dates are listed in **Table 1**. **Figure 2** shows the box plots of the fresh biomass data for the Tc and Cal panels. Differences in fresh biomass across plots and between years can be attributed to the large number of hybrids in each panel, as well as to the impact of different planting dates and weather during the growing season. For example, the two panels in 2019 were planted approximately one month later than the other years due to an excessively wet spring.

### Remote sensing data

**Figure 3:**
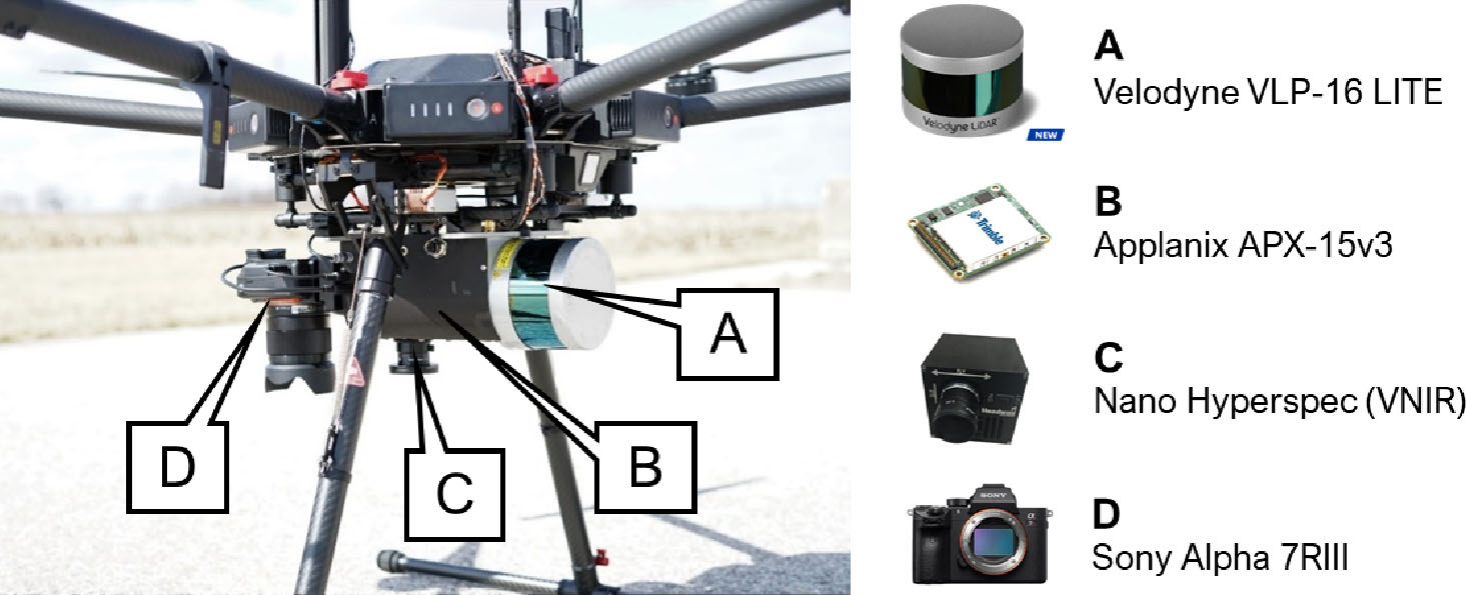
The Unmanned Aerial Vehicle (UAV) mobile mapping system and onboard sensors.

The data acquisition platform was a DJI Matrice 600 Pro (M600P) platform which carried a custom mounted Sony α7R III RGB camera, Headwall Nano-Hyperspec Visible Near Infrared (VNIR) push-broom scanner, and Velodyne VLP-16 Puck Lite laser unit (**Figure 3**). RGB data products were not used in this study. The hyperspectral scanner has 272 spectral bands with a wavelength range from 400 nm to 1000 nm and a pixel pitch of 7.4 μm. The LiDAR unit has a range accuracy of ±3 cm with a maximum range of 100 m. Georeferencing information was collected by a Trimble APX-15 UAV v3 integrated Global Navigation Satellite Systems/Inertial Navigation Systems (GNSS/INS) unit. The expected post-processing positional accuracy is ±2 cm to ±5 cm, and the attitude accuracy is ±0.025˚ and ±0.08˚ for the roll/pitch and heading, respectively. Rigorous system calibration was performed for direct georeferencing using the procedure described in Ravi et al. (2018).

The RS data used in this paper were acquired by the hyperspectral scanner and LiDAR unit onboard the UAV during the growing season in each year. The flying height of all the flight missions was ~44 m, which was designed to ensure a 4 cm Ground Sampling Distance (GSD) for the hyperspectral scanner. Four RS datasets were selected for each trial based on the Growing Degree Days (GDDs) to represent different growing stages, as shown in **Table 2**. The hyperspectral data were converted to reflectance using calibrated reference targets and the empirical line method (Ortiz-Jiménez et al., 2020), orthorectified (Habib et al., 2018), and mosaicked. Examples of the hyperspectral orthomosaic and reconstructed LiDAR point cloud are shown in **Supplementary Figure 1**. The weather data, including temperature and precipitation, were provided by the Midwestern Regional Climate Center (station name: West Lafayette 6 NW). The daily solar radiation data was collected by an Eppley solar sensor located at ACRE. The genetic marker data were provided by the breeder.

**Table 2:** Remote sensing data sets used in this study.

Trial	Year	Data Type	Dates			
*Tc*	2018	VNIR	06/14	07/06	07/25	08/02
		LiDAR	06/11	07/02	07/23	08/01
*Tc*	2019	VNIR	07/12	07/23	08/10	08/24
		LiDAR				
*Cal*	2019	VNIR	07/12	07/23	08/10	08/24
		LiDAR				
*Tc*	2020	VNIR	06/19	07/08	07/25	08/06
		LiDAR				07/28
*Cal*	2020	VNIR	06/17	07/08	07/25	08/06
		LiDAR				07/28
*Cal*	2021	VNIR	07/03	07/19	08/02	08/16
		LiDAR				

### Feature extraction and importance analysis

Both hyperspectral and LiDAR features were extracted from the rows 2 and 3 in each plot (from hyperspectral orthomosaics and LiDAR point cloud data) to minimize the border effects (**Supplementary Figure 2**). The candidate hyperspectral features include vegetation indices, integration features, and derivative features (Masjedi et al., 2020). Integration features are areas under the mean spectral curves over defined ranges of wavelength. Derivative features include first and second derivatives of the spectral signatures. Candidate LiDAR features include different percentiles of height, canopy volume, multiple statistical features, and LiDAR canopy cover (Masjedi, 2020; Masjedi et al., 2020). The percentiles of height, canopy volume, and multiple statistical values were calculated based on the non-ground points of rows 2 and 3 in each plot. The LiDAR canopy cover is defined as a ratio of the number of points above a certain height and the total number of points within the respective row segment. Weather features include cumulative precipitation, radiation, and GDDs (shown in **Supplementary Figure 3**).

Redundancy in the features slows the training process and reduces the robustness of deep learning neural networks (Ortiz-Jiménez et al., 2020). In this paper, the importance of the hyperspectral and LiDAR features is evaluated, and those which are less important are removed from the prediction model. First, a principal component analysis (PCA) is applied to an $N_{p}\;\times N_{f}$ matrix, where $N_{p}$ is the number of plots and $N_{f}$ is the number of features. The importance of a feature, $I_{f_{i}}$ , is calculated as Eq. (1), where ${\left(W_{f_{i}}\right)}_{n}$ is the magnitude of feature i in the $n_{th}$ principal component (PC) and $V_{n}$ is the variance explained by the $n_{th}$ PC. For a single trial, feature importance $I_{f_{i}}$ is evaluated for each of the selected RS datasets, as the importance of a feature could change over time. Features with lower importance across time are then identified. For multiple field trials, the above-mentioned procedure is repeated for each trial, and majority voting is used to select the common redundant features. In this study, features with lower importance, identified in more than half number of the trials, were selected and removed from the feature list.


(1) $$I_{f_{i}}={\left(W_{f_{i}}\right)}_{n}*V_{n}$$

The genetic marker data are extraordinarily high dimensional, as each hybrid is represented as a 1 × 80,104 vector. To reduce its dimensionality of genetics, PCs of the original genetic marker data are computed, and a small number (based on the variance explained) of components are selected (in this study, the first 10 PCs are used). A *k*-means clustering is then performed on the projected marker data. To determine the number of clusters, *k*, the within-cluster sum of squares (WCSS), which measures the variability of the data within each cluster, is calculated for different *k* values. The Elbow method that plots the WCSS against the *k* values is utilized to identify the optimal *k* value. The resulting genotype clusters serve as the genetic information extracted from the high dimensional genetic maker data and can be directly used together with other features in the neural network.

### LSTM-based RNN-G model

**Figure 4:**
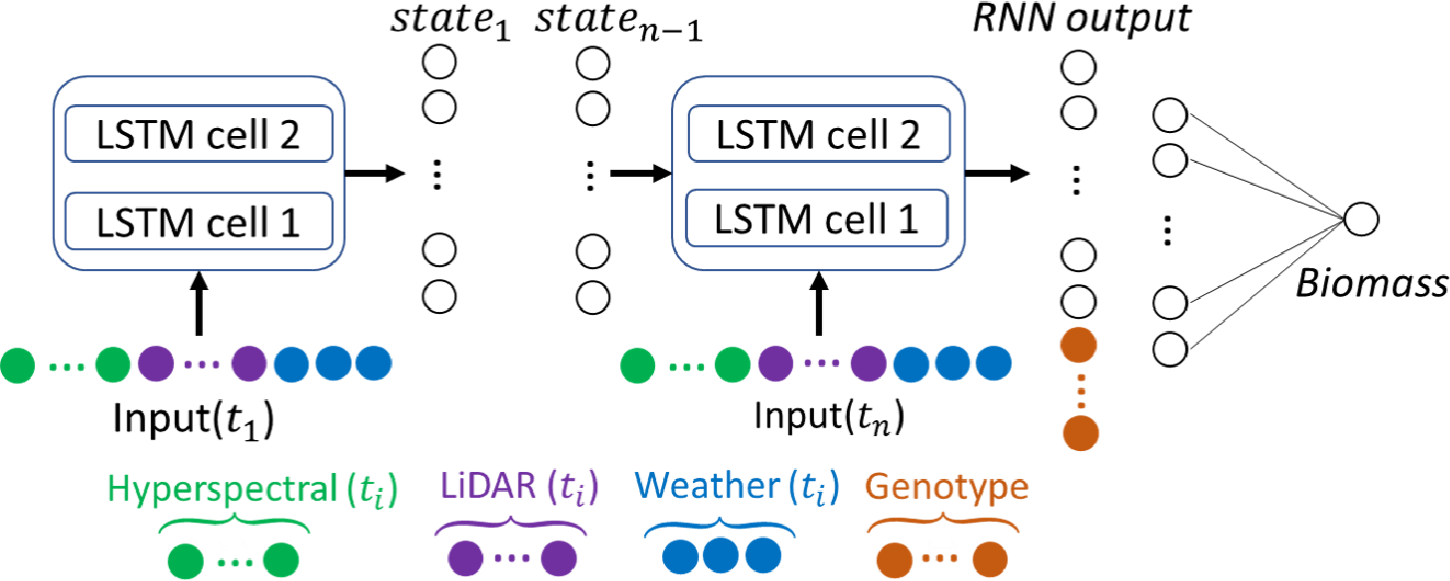
RNN-G biomass prediction model and input vectors including hyperspectral, LiDAR, and weather features at each stage, and the genotype clusters.


To efficiently use both time-series features (RS and weather) and static feature (genetic marker clusters), an LSTM-based RNN model (architecture in **Figure 4**), referred to as RNN-G, is proposed. Different numbers of stacked LSTM-cells were explored based on the experimental data, and the sensitivity analysis indicated 2 is the optimal number. RS and weather features are calculated at each date and used as input vectors of the stacked LSTM-cells. In this study, four RS datasets were selected throughout the growing season based on their GDDs, representing different stages of plant development. The input dimensionality of the RNN-G model is $N_{f}\times N_{t}$ where $N_{f}$ is the number of RS and weather features, and $N_{t}$ is the number of time steps (in this study $N_{t}=4)$ . To avoid redundant parameters in the prediction model, the static genotype clusters and the output of the stacked LSTM-cells are concatenated and used as input of a fully connected layer. The Mean Squared Error (MSE) loss function was used as a metric in fitting the model. The Adam optimizer (Kingma and Ba, 2014) was used to update the network weights and minimize the loss function during the training process. The RNN-G model was trained on each set of trial using 3-fold cross validation. One-third of the plots were randomly selected as the test set, and the remaining plots were split into 90% as training set and 10% as a validation set (to allow adequate data for training). The modeling strategy needed to accommodate both the limited quantity of reference data and the diversity across the experiment. An independent test set was not selected for these reasons. To evaluate the performance of the model, the $R_{ref}^{2}$ was calculated as per Eq. (2). Here, $y_{i}$ and $\hat{y_{i}}$ represent the ground reference and predicted fresh biomass of plot i , respectively.

(2) $$R_{ref}^{2}=1-\;\frac{\sum_{i}{\left(y_{i}-\;\hat{y_{i}}\right)}^{2}}{\sum_{i}{\left(y_{i}-\bar{y}\right)}^{2}}$$

### Transfer learning strategies

In breeding programs, numerous phenotypic traits are measured in-field at different growing stages, which, as noted previously, is traditionally labor- and time-consuming. For example, an experimental panel with a large number of testcross hybrids (the *Tc* panel in this study) usually cannot be destructively sampled multiple times during the growing season due to resource constraints. To address this issue, breeders select representative hybrids and plant them in a small field referred to as the calibration panel (e.g. *Cal* panel in this study). With a relatively small number of hybrids, destructive sampling can occur multiple times during the growing season, providing a time series of ground reference data for the LSTM-based RNN prediction model. A key objective of this study is to develop transfer learning strategies for the model trained with RS data acquired over the *Cal* panel, and perform biomass prediction on the larger *Tc* panel. The pre-trained model is fine-tuned with limited training samples and used to perform prediction in the target domain, which contains many hybrids that are unseen in the source domain. Two transfer learning strategies for identifying optimal training samples from the target domain are investigated: the *genomic* strategy and the *phenotype* strategy. The hybrids manually selected by breeders and planted in the *Cal* panel are assumed to be broadly representative. Thus, the *genomic* strategy selects the same hybrids that are in the *Cal* panel from the *Tc* panel, and fine-tunes the pre-trained model to accommodate local differences. The *phenotype* strategy, as described in (Wang and Crawford, 2021), assumes that the extracted RS features represent phenotypes that are highly correlated with biomass. By clustering the RS features, the algorithm selects the most representative training samples from the phenotypic point of view. The *k*-means clustering algorithm is applied to the RS features extracted from the *Tc* panel. The suitable number of clusters is identified, and the samples with the smallest distance to the centers of each cluster are selected for fine-tuning the pre-trained model.

## Experimental results and discussion

### Remote sensing feature importance analysis

**Figure 5:**
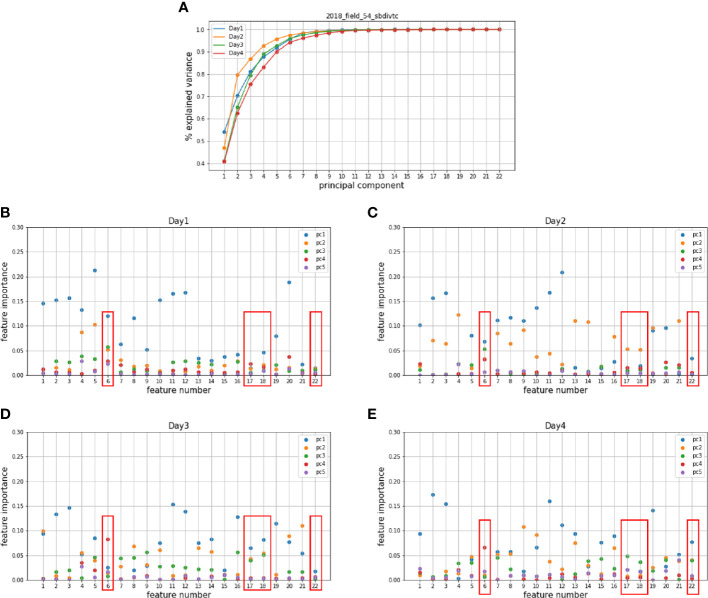
Principal components of the hyperspectral features in **(A)** 2018 *Tc* panel and the corresponding importance of hyperspectral features on **(B)** the 1st, **(C)** the 2nd, **(D)** the 3rd, **(E)** the 4th dates which represents four different growing stages.

**Figure 6:**
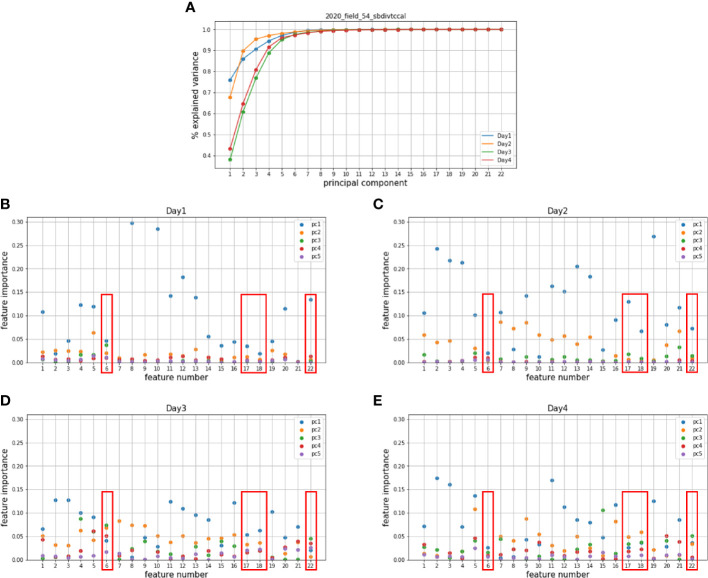
Principal components of the hyperspectral features in **(A)** the 2020 Cal panel and the corresponding importance of all hyperspectral features on **(B)** the 1st, **(C)** the 2nd, **(D)** the 3rd, **(E)** the 4th dates which represent four different growing stages.

The proposed feature importance analysis was applied to the hyperspectral and LiDAR features from the *Tc* and *Cal* panels in 2018, 2019, and 2020. The original hyperspectral and LiDAR features are listed in **Supplementary Table 1** and **Supplementary Table 2** (Masjedi, 2020; Masjedi et al., 2020). The hyperspectral features were evaluated at plot level, resulting in an $N_{p}\;\times 22$ feature matrix for each data set, where $N_{p}$ denotes the number of plots. PCs were extracted from the individual data sets, and the variance of 22 PCs are shown in **Figures 5A**, **6A**. The first 5 PCs explained more than 90% of the variance in all the individual dates across growing season. The feature importance ( $I_{f_{i}}$ ) based on the first 5 PCs for the *Tc* and *Cal* panels is shown in **Figures 5B–E** and **Figures 6B–E**, respectively. As illustrated in the figures, different features are dominant within different PCs over the season. For example, for date 1 of the 2018 *Tc* panel, the first PC was dominated by features 5 and 20; the second PC was dominated by features 4 and 5. The dominant features change over time or across experimental trials based on the plant physiology and the local environmental conditions. In this study, a feature is considered redundant only if it has low importance values at all growth stages within a year. Common redundant features among all experimental trials across years were identified by majority voting. The experimental results are quite consistent for the same trials planted in different years. Three vegetation indices and one derivative feature (6^th^, 17^th^, 18^th^, and 22^nd^ features) were identified as redundant features and removed from the prediction model.

**Figure 7:**
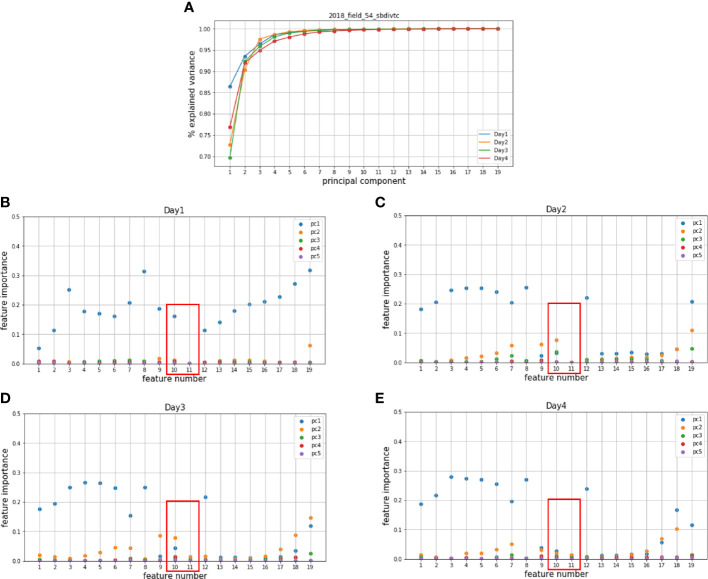
Principal components of the LiDAR features in **(A)** 2018 *Tc* panel and the corresponding importance of all LiDAR features on **(B)** 1st, **(C)** 2nd, **(D)** 3rd, **(E)** 4th dates which represents 4 different growing stages.

**Figure 8:**
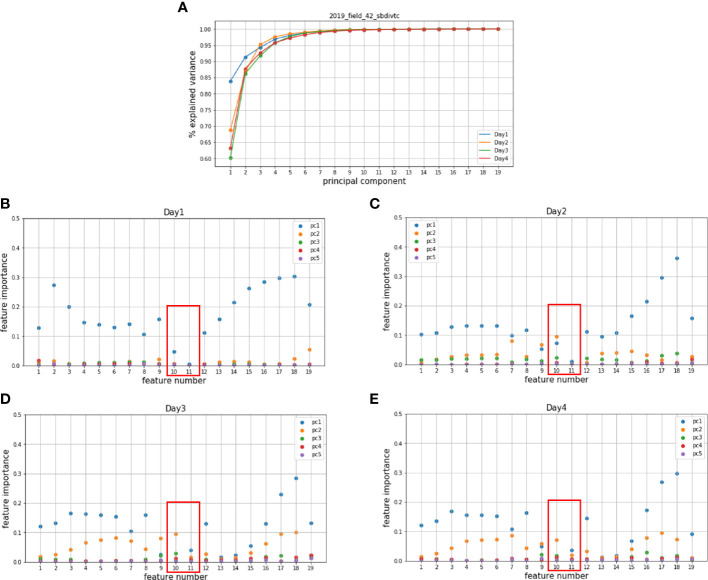
Principal components of the LiDAR features in **(A)** 2019 *Tc* panel and the corresponding importance of all LiDAR features on **(B)** 1st, **(C)** 2nd, **(D)** 3rd, **(E)** 4th dates which represents 4 different growing stages.

Similar to the hyperspectral features, the original LiDAR features resulted in an $N_{p}$  ×19 feature matrix, where $N_{p}$ denotes the number of plots. The variance explained by each PC is shown in **Figure 7A** and **Figure 8A**; the first 5 PCs explained more than 95% variance in the 4 dates across the growing season. **Figures 7B–E**, **8B–E** show the results of the importance analysis for the *TC* and *Cal* panels, respectively. The results suggest that different features can be critical at different growing stages or environments. The 10^th^ and 11^th^ LiDAR based features were identified as redundant among all experimental trials across years and thus were removed from the prediction model.

### Genotype clusters based on genetic markers

**Figure 9:**
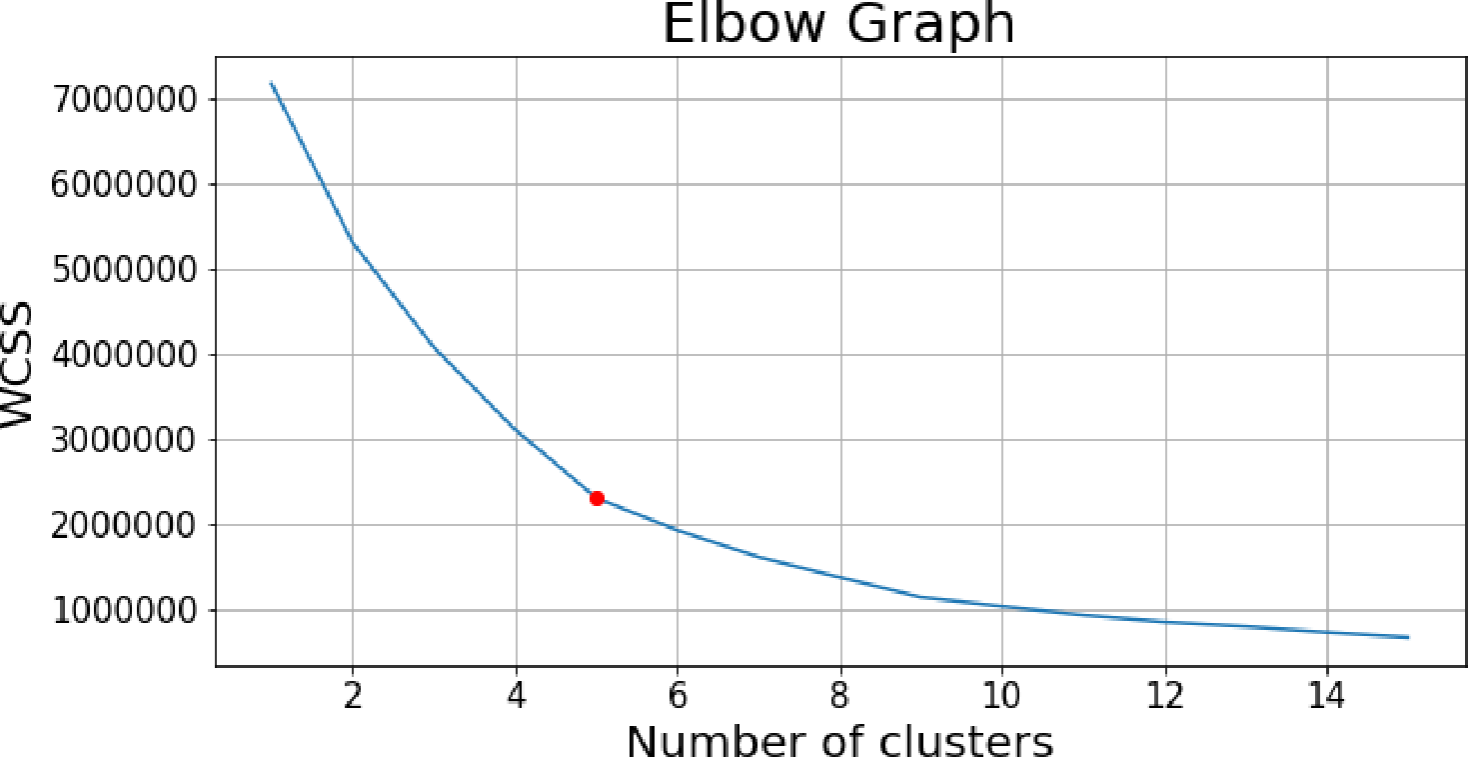
Elbow point graph of within-cluster sum of squares, the red point shows the selected number of clusters.

PCs of the genetic marker data are of dimension $N_{v}\;\times 80,\;104$ , where $N_{v}$ is the number of hybrids. The optimal number of clusters was determined experimentally, as noted previously. The WCSS decreases rapidly as the number of clusters *k* increases at the beginning. The optimal value of *k* was selected at the curve of the elbow graph. In this study, the elbow graph shown as **Figure 9** suggests the number of clusters to be 5. A visualization of the 5 clusters is shown in **Supplementary Figure 4**. **Supplementary Figures 4A, B** show different combinations of 3 principal components to visualize the 5 clusters in 3D plots. Each color represents a unique cluster. Based on the results, hybrids belonging to the same category were grouped together. The cluster ID serves as an input feature representing the genetic information in the prediction model.

### Impact of incorporating genetic information

To illustrate the impact of incorporating genetic information into the sorghum biomass prediction model, results from the *Tc* panels in 2018, 2019, and 2020 are included in this section. The learning rate was determined experimentally and set to 0.0005 with the Adam optimizer, and the number of epochs was set to 1000. The end-of-season fresh biomass of individual plots was the predicted variable. Prediction performance of the LSTM-based RNN model was evaluated based on the difference between the predicted and ground reference values ( $R_{ref}^{2}$ ). A negative $R_{ref}^{2}$ indicates a bias in the predicted values. Two models were developed:

- Model A: single year biomass prediction using RS and weather data only;
- Model B: single year biomass prediction using RS, weather, and genotype cluster information.

**Figure 10:**
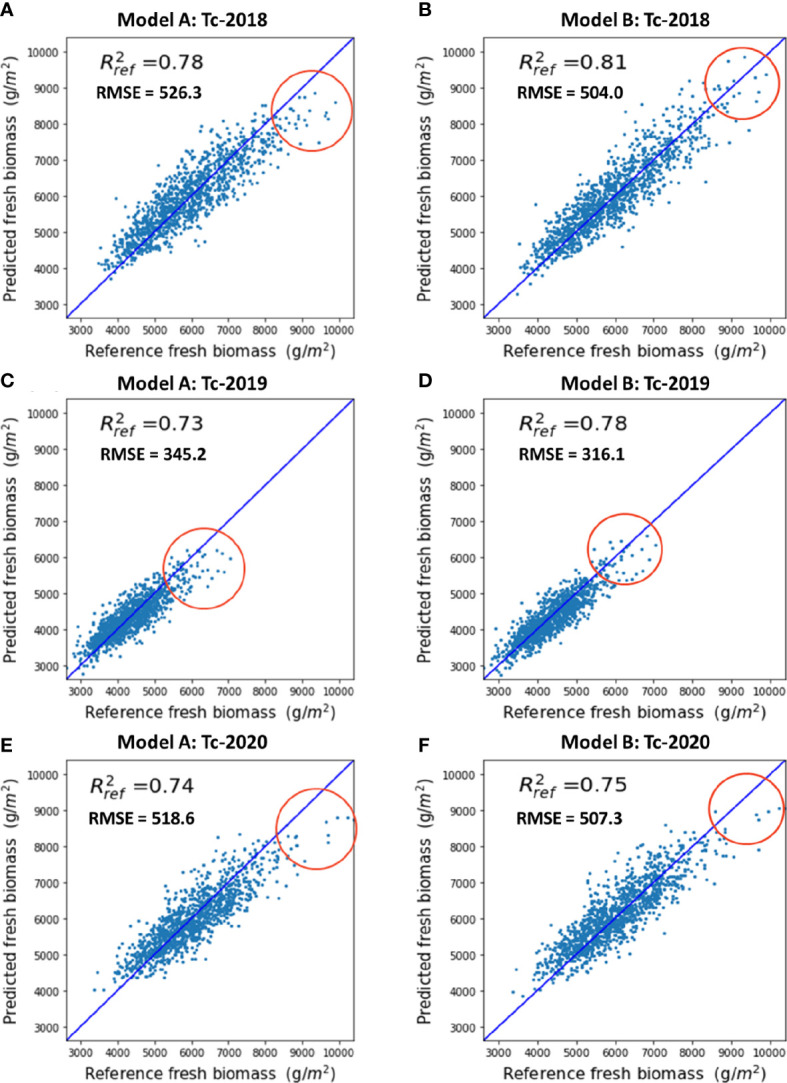
Performance of single year biomass prediction: **(A)** Model A on the 2018 *Tc* panel, **(B)** Model B on the 2018 *Tc* panel, **(C)** Model A on the 2019 *Tc* panel, **(D)** Model B on the 2019 *Tc* panel, **(E)** Model A on the 2020 *Tc* panel, **(F)** Model B on the 2020 *Tc* panel. Red circles show the improvement of the prediction results with genetic information, which occurred for high biomass reference values, typically associated with photoperiod sensitive hybrids.

The prediction performance of Models A and B was evaluated against the ground reference data, as shown in **Figure 10**. As can be seen in **Figures 10A, C**, **E**, Model A tended to under-predict the fresh biomass for the high-yielding plots (as marked by the red circles). By incorporating the genotype information, Model B was able to provide a more reliable estimate of the end-of-season fresh biomass for these high-yielding hybrids, as shown in **Figures 10B, D**, **F**.

### Transfer learning from *Cal* to *Tc*

The two proposed transfer learning strategies were evaluated using the *Tc* and *Cal* panels in 2018, 2019, 2020, and 2021. Results of two scenarios were investigated: i) the *Tc* and *Cal* panels were from different fields in the same year, and ii) the *Tc* and *Cal* panels were from different years.

The *Tc* and *Cal* panels in 2019 and 2020 were used for the first scenario. As mentioned in Section 3.1, the *Cal* panels contain 72 hybrids which are a subset of 630 hybrids in the *Tc* panels. Four models were developed based on *Tc* and *Cal* panels in 2019 (**Table 3**):

- Model R19tc: the RNN-G pre-trained with the *Tc* panel in 2019;
- Model R19cal: the RNN-G pre-trained with the *Cal* panel in 2019;
- Model R19cal-G19: Model R19cal fine-tuned with training samples selected by the *genomic* strategy from the *Tc* panel in 2019;
- Model R19cal-P19: Model R19cal fine-tuned with training samples selected by the *phenotype* strategy from the *Tc* panel in 2019.

**Table 3:** Biomass prediction results obtained by the 4 models on the *Tc* and *Cal* panels in 2019.

Model	R19tc (pre-trained with Tc-2019)	R19cal (pre-trained with Cal-2019)	R19cal-G19 (fine-tuned R19tc with genomic strategy)	R19cal-P19 (fine-tuned R19tc with phenotype strategy)
Number of training samples from 2019 *Tc* panel	726	0	129	70
Number of training hybrids from 2019 *Tc* panel	378	0	67	68
$R_{ref}^{2}$ of prediction	0.78	-5.02	0.66	0.62
RMSE of prediction ( $g/m^{2}$ )	316.14	1703.64	397.43	410.59
$R_{ref}^{2}$ of ranking	0.81	0.54	0.69	0.65

**Table 3** shows the results obtained with all four models over the *Tc* panel in 2019, including the number of training samples/hybrids, $R_{ref}^{2}$ and root-mean-square error (RMSE) values of prediction, and $R_{ref}^{2}$ values of ranking. The average fresh biomass weights of each hybrid were used to calculate the $R_{ref}^{2}$ values of ranking. Both models R19cal and R19tc were used as baseline models. Model R19tc was trained with target domain data (*Tc* panel) and thus was expected to have the highest $R_{ref}^{2}$ value and the lowest RMSE value. Model R19cal was trained with source domain data (*Cal* panel) without using any information from the target domain (*Tc* panel), and thus yielded the lowest $R_{ref}^{2}$ value and the highest RMSE value. Based on **Table 3**, models R19cal-G19 and R19cal-P19 achieved similar $R_{ref}^{2}$ values in both prediction and ranking. The two models also obtained similar RMSE values in prediction. Compared to model R19cal, both the *genomic* and *phenotype* strategies achieved significant improvement of prediction and ranking results. It should also be noted that the *phenotype* strategy (R19cal-P19) required only about half the quantity of training data used by the *genomic* strategy (R19cal-G19). However, both strategies have nearly the same number of hybrids. As mentioned previously, the *Cal* panel contains a subset of genotypes manually selected by the breeder according to his belief that these hybrids are representative of the 630 genotypes planted in *Tc*. The conceptual basis of the *phenotype* strategy is that *k*-means clustering can effectively represent the phenotypic groups and identify informative training samples. This result implies that from both genotypic and phenotypic points of view, the variation/behavior of the 630 genotypes in the *Tc* panel can be represented by the around 70 genotypes.

Both the *genomic* and *phenotype* strategies selected a similar number of hybrids, and the selected hybrids cover the five major genotype clusters (**Supplementary Figure 5**). The PC representation of hybrids based on the *phenotype* strategy is essentially the same as for the *genomic* strategy.

The analysis was repeated to investigate consistency. Four models were developed based on *Tc* and *Cal* panels planted in 2020 (**Table 4**):

- Model R20tc: the RNN-G pre-trained with *Tc* panel in 2020;
- Model R20cal: the RNN-G pre-trained with *Cal* panel in 2020;
- Model R20cal-G20: Model R20cal, fine-tuned with training samples selected by the *genomic* strategy from the *Tc* panel in 2020;
- Model R20cal-P20: Model R20cal, fine-tuned with training samples selected by the *phenotype* strategy from the *Tc* panel in 2020.

**Table 4:** Biomass prediction results obtained by the 4 models on the *Tc* and *Cal* panels in 2020.

Model	R20tc (pre-trained with Tc-2020)	R20cal (pre-trained with Cal-2020)	R20cal-G20 (fine-tuned R20tc with genomic strategy)	R20cal-P20 (fine-tuned R20tc with phenotype strategy)
Number of training samples from 2020 *Tc* panel	744	0	143	80
Number of training hybrids from 2020 *Tc* panel	417	0	72	77
$R_{ref}^{2}$ of prediction	0.76	0.07	0.68	0.67
RMSE of prediction ( $g/m^{2}$ )	504.21	933.11	571.93	582.27
$R_{ref}^{2}$ of ranking	0.77	0.58	0.72	0.71

The fresh biomass prediction performance of the four models over the *Tc* panel in 2020 is shown in **Table 4**. Models R20cal and R20tc were used as baseline models to show the best and worst performances, respectively. Models R20cal-G20 and R20cal-P20 achieved similar $R_{ref}^{2}$ values in prediction and ranking, and they also had similar RMSE values in prediction. The performances of Models R20cal-G20 and R20cal-P20 showed significant improvement compared to Model R20cal. In fact, their performances were quite comparable to the Model R20tc. As in 2019, both the *genomic* and *phenotype* strategies selected a similar number of hybrids from the five major genotype clusters (**Supplementary Figure 6**). Overall, the findings of analyses conducted on *Tc* and *Cal* panels in 2019 and 2020 are consistent. The results show that the proposed transfer learning strategies can efficiently select critical training samples and achieve reasonable prediction results over the *Tc* panel while using less than 20% of the training data.

In the second scenario, the *Tc* and *Cal* panels planted in different years (2018 and 2021) were used in an experimental trial. The different environmental conditions associated with the field and weather between the two years provided more challenging conditions to verify the robustness of the transfer learning strategies. Similarly, four models were developed to verify the performance of the two proposed transfer learning strategies (**Table 5**):

- Model R18tc: the RNN-G pre-trained with the *Tc* panel in 2018;
- Model R21cal: the RNN-G pre-trained with the *Cal* panel in 2021;
- Model R21cal-G18: Model R21cal fine-tuned with training samples selected by the *genomic* strategy from the *Tc* panel in 2018;
- Model R21cal-P18: Model R21cal fine-tuned with training samples selected by the *phenotype* strategy from the *Tc* panel in 2018.

**Table 5:** Biomass prediction results obtained by the 4 models on the *Tc* panel in 2018 and *Cal* panel in 2021.

Model	R18tc (pre-trained with Tc-2018)	R21cal (pre-trained with Cal-2021)	R21cal-G18 (fine-tuned R18tc with genomic strategy)	R21cal-P18 (fine-tuned R18tc with phenotype strategy)
Number of training samples from 2018 *Tc* panel	732	0	131	80
Number of training hybrids from 2018 *Tc* panel	419	0	67	76
$R_{ref}^{2}$ of prediction	0.81	0.57	0.69	0.69
RMSE of prediction ( $g/m^{2}$ )	504.03	748.85	633.07	638.14
$R_{ref}^{2}$ of ranking	0.82	0.58	0.70	0.70

**Table 5** shows the evaluation results over the *Tc* panel in 2018 for the four models. Models R21cal and R18tc were used as baseline models. According to **Table 5**, the proposed two transfer learning strategies, Models R21cal-G18 and R21cal-P18, achieved similar $R_{ref}^{2}$ values in prediction and ranking. The two models also obtained similar RMSE values in prediction. Compared with the lowest reference value, Model R21cal, both transfer learning strategies obtained higher $R_{ref}^{2}$ values and lower RMSE values, and their performances were quite comparable to the highest reference, Model R18tc. The number of hybrids selected by the *genomic* and *phenotype* strategies are similar, and these hybrids cover the five major genotype clusters (**Supplementary Figure 7**).

In summary, both the *genomic* and *phenotype* strategies significantly improved the performance of the pre-trained model in the target domain in both experiments in the same year (weather conditions equivalent) and different years (different fields and different weather). The results also indicate that the *phenotype* strategy can achieve similar prediction performance as the *genomic* strategy using only half number of the training samples.

### Summary of experimental results

In section *Remote sensing feature importance analysis*, based on the remote sensing feature importance analysis results from three years’ *Tc* and *Cal* panels, 4 hyperspectral and 2 LiDAR features were identified as redundant features and removed from the prediction model.

In section *Genotype clusters based on genetic markers*, the unsupervised genotype clustering result showed all the hybrids involved in this study (630) can be grouped into 5 major clusters. The cluster IDs were used as input information for the RNN-G biomass prediction model.

In section *Impact of incorporating genetic information*, the advantage of incorporating the genetic information into the biomass prediction model was demonstrated. Model B (with genetic information) achieved more accurate prediction results on the high-yielding plots than Model A (without genetic information).

In section *Transfer learning from Cal to Tc*, the two proposed transfer learning strategies were verified under two scenarios:

- The *Tc* and *Cal* panels were from different fields in the same years: the experiments were conducted in two years (2019 and 2020) to investigate consistency. Models R19cal-G19/R20cal-G20 and R19cal-P19/R20cal-P20 (using *genomic* and *phenotype* strategies) obtained significant improvement over the lowest baseline models, respectively.
- The *Tc* and *Cal* panels were from different years: the Tc-2018 and Cal-2021 were analyzed. With different environmental conditions, models R21cal-G18 and R21cal-P18 (using *genomic* and *phenotype* strategies) still achieved comparable prediction results to the highest baseline model, respectively.

Overall, the two transfer learning strategies had similar performance and both tended to select training samples to cover all 5 major genotype clusters.

## Conclusions and discussion

This study proposed an LSTM-based RNN model for plot-level sorghum biomass prediction using remote sensing, weather, and genotypic data. Feature importance analysis was utilized to identify remote sensing features with low importance across multiple growing stages and fields. These features were removed from the biomass prediction model to reduce the redundancy and enhance robustness of the model. The number of features removed was surprisingly small; this was primarily due to the importance of features being time dependent over the growing season. A strategy for dimension reduction of genetic marker data was implemented. The architecture of the proposed LSTM-based RNN model is designed to handle the time series remote sensing and weather data, as well as the static genetic information. Two transfer learning strategies, the *genomic* and *phenotype* strategies, were investigated to leverage the knowledge learned from a subset of hybrids in a breeding program. The performance of the proposed strategies was assessed over a sorghum breeding experiment planted in multiple years with more than 600 testcross hybrids. Experimental results showed that the hybrids could be clustered into 5 major classes based on their genotype information. The proposed LSTM-based RNN biomass prediction model achieved a high accuracy ( $R_{ref}^{2}$ value around 0.8) for single year prediction. In terms for transfer learning, both the *genomic* and *phenotype* strategies effectively selected critical training samples and thus improved the performance of the pre-trained model in the target domain. Based on the genotype cluster plots of the selected samples, the two transfer learning strategies sought to accommodate the variability of the hybrids in the target domain. This result also indicates the strong relationship between crop phenotyping and genotyping. For large crop breeding experiments, phenotyping can potentially be used as a key factor to select the most informative training samples for the biomass prediction model and help reduce the manual harvest work.

Currently, the proposed LSTM-based RNN biomass prediction model requires time-series RS data throughout the whole growing season. Use of partial growing season RS data to predict the end-of-season biomass at an early stage is being explored to provide early rankings and thus allow concentrate effort on promising hybrids.

## Data availability statement

The raw data supporting the conclusions of this article will be made available by the authors, without undue reservation.

## Author contributions

TW and MC: Conceptualization, formal analysis, and methodology. TW: Writing—original draft. MC and MT: Supervision, review, and editing. All authors have read and agreed to the published version of the manuscript. All authors contributed to the article and approved the submitted version.

## Supplementary material

Supplementary Figure 1 An example of **(A)** hyperspectral orthomosaic, and **(B)** reconstructed LiDAR point cloud over field 54.

Supplementary Figure 2 Example 4-row plot from the Tc panel in 2020; Features extracted from areas within the yellow boxes.

Supplementary Figure 3 Cumulative environmental features during 2018-2021 growing season: **(A)** cumulative precipitation, **(B)** cumulative GDDs, and **(C)** cumulative radiation.

Supplementary Figure 4 3D plots of the five genotype clusters in different colors: **(A)** plot showing *the three principal components (PCs)* PC1, PC2, and PC3, and **(B)** plot showing PC1, PC2, and PC4.

Supplementary Figure 5 Genotype clusters of the training samples selected by two transfer learning strategies, shown in 3D plots with different combinations of principal components (PCs), **(A)** shows the 67 hybrids selected by R19cal-G19 and the distribution of those hybrids in all hybrid lines; **(B)** shows the 68 hybrids selected by R19cal-P19 and the distribution of those hybrids in all hybrid lines.

Supplementary Figure 6 Genotype clusters of the training samples selected by two transfer learning strategies, shown in 3D plots with different combinations of principal components (PCs), **(A)** shows the 72 hybrids selected by R20cal-G20 and the distribution of those hybrids in all hybrid lines; **(B)** shows the 77 hybrids selected by R20cal-P20 and the distribution of those hybrids in all hybrid lines.

Supplementary Figure 7 Genotype clusters of the training samples selected by two transfer learning strategies, shown in 3D plots with different combinations of principal components (PCs), **(A)** shows the 67 hybrids selected by R21cal-G18 and the distribution of those hybrids in all hybrid lines; **(B)** shows the 76 hybrids selected by R21cal-P18 and the distribution of those hybrids in all hybrid lines.

## References

[B1] Abdalla A. Cen H. Wan L. Rashid R. Weng H. Zhou W.. (2019). Fine-tuning convolutional neural network with transfer learning for semantic segmentation of ground-level oilseed rape images in a field with high weed pressure. Comput. Electron Agric. 167, 105091. doi: 10.1016/j.compag.2019.105091

[B2] Altman N. Krzywinski M. (2018). The curse(s) of dimensionality. Nat. Methods 15, 399–400. doi: 10.1038/s41592-018-0019-x 29855577

[B3] Ballesteros R. Ortega J. F. Hernandez D. Moreno M. A. (2018). Onion biomass monitoring using UAV-based RGB imaging. Precis. Agric. 19, 840–857. doi: 10.1007/s11119-018-9560-y

[B4] Cai E. Baireddy S. Yang C. Crawford M. Delp E. J. (2020). Deep transfer learning for plant center localization. In Proceedings of the IEEE/CVF conference on computer vision and pattern recognition workshops. 62–63. doi: 10.1109/CVPRW50498.2020.00039

[B5] Caruana R. Pratt L. Thrun S. (1997). Multitask learning. Mach. Learn 28, 41–75. doi: 10.1023/A:1007379606734

[B6] Chao Z. Liu N. Zhang P. Ying T. Song K. (2019). Estimation methods developing with remote sensing information for energy crop biomass: a comparative review. Biomass Bioenergy 122, 414–425. doi: 10.1016/j.biombioe.2019.02.002

[B7] Comba L. Biglia A. Ricauda Aimonino D. Tortia C. Mania E. Guidoni S.. (2020). Leaf area index evaluation in vineyards using 3D point clouds from UAV imagery. Precis. Agric. 21, 881–896. doi: 10.1007/s11119-019-09699-x

[B8] Dai W. Xue G.-R. Yang Q. Yu Y. (2007). “Transferring naive bayes classifiers for text classification,” in Association for the advancement of artificial intelligence. 1, 540–545. Available at: www.aaai.org.

[B9] Dong X. Chowdhury S. Qian L. Li X. Guan Y. Yang J.. (2019). Deep learning for named entity recognition on Chinese electronic medical records: combining deep transfer learning with multitask bi-directional LSTM RNN. PloS One 14 (5), e0216046. doi: 10.1371/journal.pone.0216046 31048840 PMC6497281

[B10] Fan J. Bai J. Li Z. Ortiz-Bobea A. Gomes C. P. (2022) A GNN-RNN approach for harnessing geospatial and temporal information: application to crop yield prediction. Available at: www.aaai.org.

[B11] Fassnacht F. E. Hartig F. Latifi H. Berger C. Hernández J. Corvalán P.. (2014). Importance of sample size, data type and prediction method for remote sensing-based estimations of aboveground forest biomass. Remote Sens Environ. 154, 102–114. doi: 10.1016/j.rse.2014.07.028

[B12] Feng L. Chen S. Zhang C. Zhang Y. He Y. (2021). A comprehensive review on recent applications of unmanned aerial vehicle remote sensing with various sensors for high-throughput plant phenotyping. Comput. Electron Agric. 182, 106033. doi: 10.1016/j.compag.2021.106033

[B13] Feng P. Wang B. Liu D. L. Waters C. Xiao D. Shi L.. (2020). Dynamic wheat yield forecasts are improved by a hybrid approach using a biophysical model and machine learning technique. Agric. For Meteorol. 285–286, 107922. doi: 10.1016/j.agrformet.2020.107922

[B14] Gers F. A. Schmidhuber J. Cummins F. (2000). Learning to forget: Continual prediction with LSTM. Neural Comput. 12 (10), 2451–2471. doi: 10.1162/089976600300015015 11032042

[B15] Gong L. Yu M. Jiang S. Cutsuridis V. Pearson S. (2021). Deep learning based prediction on greenhouse crop yield combined TCN and RNN. Sensors 21 (13), 4537. doi: 10.3390/s21134537 34283083 PMC8271501

[B16] Graves A. (2013) Generating sequences with recurrent neural networks. Available at: http://arxiv.org/abs/1308.0850.

[B17] Graves A. Jaitly N. Mohamed A. R. (2013). “Hybrid speech recognition with deep bidirectional LSTM,” in IEEE Workshop on Automatic Speech Recognition and Understanding. 273–278. doi: 10.1109/ASRU.2013.6707742

[B18] Graves A. Wayne G. Danihelka I. (2014) Neural turing machines. Available at: http://arxiv.org/abs/1410.5401.

[B19] Habib A. Zhou T. Masjedi A. Zhang Z. Evan Flatt J. Crawford M. (2018). “Boresight calibration of GNSS/INS-assisted push-broom hyperspectral scanners on UAV platforms,” in IEEE J Sel Top Appl Earth Obs Remote Sens. 11, 1734–1749. doi: 10.1109/JSTARS.2018.2813263

[B20] Hammer G. L. van Oosterom E. McLean G. Chapman S. C. Broad I. Harland P.. (2010). Adapting APSIM to model the physiology and genetics of complex adaptive traits in field crops. J. Exp. Bot. 61 (8), 2185–2202. doi: 10.1093/jxb/erq095 20400531

[B21] Han L. Yang G. Dai H. Xu B. Yang H. Feng H.. (2019). Modeling maize above-ground biomass based on machine learning approaches using UAV remote-sensing data. Plant Methods 15 (1), 1–19. doi: 10.1186/s13007-019-0394-z 30740136 PMC6360736

[B22] Huntington T. Cui X. Mishra U. Scown C. D. (2020). Machine learning to predict biomass sorghum yields under future climate scenarios. Biofuels Bioprod. Biorefining 14, 566–577. doi: 10.1002/bbb.2087

[B23] Jeong S. Ko J. Yeom J. M. (2022). Predicting rice yield at pixel scale through synthetic use of crop and deep learning models with satellite data in south and north Korea. Sci. Total Environ. 802, 149726. doi: 10.1016/j.scitotenv.2021.149726 34464811

[B24] Jia Y. Shelhamer E. Donahue J. Karayev S. Long J. Girshick R.. (2014). “Caffe: Convolutional architecture for fast feature embedding,” in Proceedings of the 22nd ACM international conference on Multimedia. 675–678. doi: 10.1145/2647868.2654889

[B25] Jiang J. Zhai C. (2007). “Instance weighting for domain adaptation in NLP,” in Proceedings of the 45th annual meeting of the association of computational linguistics. 264–271. Available at: https://ink.library.smu.edu.sg/sis_research/1253.

[B26] Karami A. Crawford M. Delp E. J. (2020). “Automatic plant counting and location based on a few-shot learning technique,” in IEEE J Sel Top Appl Earth Obs Remote Sens, 13, 5872–5886. doi: 10.1109/JSTARS.2020.3025790

[B27] Kaya A. Keceli A. S. Catal C. Yalic H. Y. Temucin H. Tekinerdogan B. (2019). Analysis of transfer learning for deep neural network based plant classification models. Comput. Electron Agric. 158, 20–29. doi: 10.1016/j.compag.2019.01.041

[B28] Khaki S. Wang L. Archontoulis S. V. (2020). A CNN-RNN framework for crop yield prediction. Front. Plant Sci. 10. doi: 10.3389/fpls.2019.01750 PMC6993602 32038699

[B29] Khalid S. Khalil T. Nasreen S. (2014). “A survey of feature selection and feature extraction techniques in machine learning,” in Proceedings of 2014 Science and Information Conference. 372–378. doi: 10.1109/SAI.2014.6918213

[B30] Kingma D. P. Ba J. (2014) Adam: A method for stochastic optimization. Available at: http://arxiv.org/abs/1412.6980.

[B31] Li N. Hao H. Gu Q. Wang D. Hu X. (2017). A transfer learning method for automatic identification of sandstone microscopic images. Comput. Geosci. 103, 111–121. doi: 10.1016/j.cageo.2017.03.007

[B32] Lu D. (2006). The potential and challenge of remote sensing-based biomass estimation. Int. J. Remote Sens 27, 1297–1328. doi: 10.1080/01431160500486732

[B33] Masjedi A. (2020). Multi-temporal multi-modal predictive modelling of plant phenotypes (West Lafayette, Indiana: Thesis, Purdue University Graduate School). doi: 10.25394/PGS.12229862.v1

[B34] Masjedi A. Carpenter N. R. Crawford M. M. Tuinstra M. R. (2019). “Prediction of sorghum biomass using UAV time series data and recurrent neural networks,” in IEEE Computer Society Conference on Computer Vision and Pattern Recognition Workshops. 0–0. doi: 10.1109/CVPRW.2019.00327

[B35] Masjedi A. Crawford M. M. Carpenter N. R. Tuinstra M. R. (2020). Multi-temporal predictive modelling of sorghum biomass using UAV-based hyperspectral and lidar data. Remote Sens (Basel) 12, 1–35. doi: 10.3390/rs12213587

[B36] Messina C. D. Technow F. Tang T. Totir R. Gho C. Cooper M. (2018). Leveraging biological insight and environmental variation to improve phenotypic prediction: Integrating crop growth models (CGM) with whole genome prediction (WGP). Eur. J. Agron. 100, 151–162. doi: 10.1016/j.eja.2018.01.007

[B37] Nazeri B. Crawford M. M. Tuinstra M. R. (2021). Estimating leaf area index in row crops using wheel-based and airborne discrete return light detection and ranging data. Front. Plant Sci. 12. doi: 10.3389/fpls.2021.740322 PMC8667472 34912353

[B38] Ortiz-Jiménez G. Modas A. Moosavi-Dezfooli S.-M. Frossard P. (2020). “Redundant features can hurt robustness to distribution shift.” In Uncertainty & Robustness in Deep Learning Workshop (ICML 2020). Available at: https://corsmal.eecs.qmul.ac.uk/resources/2020.07.17_CORSMAL_ICML_UDL_paper.pdf.

[B39] Pan S. J. Yang Q. (2010). “A survey on transfer learning,” in IEEE Trans Knowl Data Eng. 22, 1345–1359. doi: 10.1109/TKDE.2009.191

[B40] Pérez-Ruiz M. Prior A. Martinez-Guanter J. Apolo-Apolo O. E. Andrade-Sanchez P. Egea G. (2020). Development and evaluation of a self-propelled electric platform for high-throughput field phenotyping in wheat breeding trials. Comput. Electron Agric. 169, 105237. doi: 10.1016/j.compag.2020.105237

[B41] Poehlman J. M. (2013). Breeding field crops (New York: Springer Science & Business Media).

[B42] Ravi R. Lin Y. J. Elbahnasawy M. Shamseldin T. Habib A. (2018). “Simultaneous system calibration of a multi-LiDAR multicamera mobile mapping platform,” in IEEE J Sel Top Appl Earth Obs Remote Sens, Vol. 11. 1694–1714. doi: 10.1109/JSTARS.2018.2812796

[B43] Reed S. Chen Y. Paine T. van den Oord A. Eslami S. M. A. Rezende D.. (2017) Few-shot autoregressive density estimation: Towards learning to learn distributions. Available at: http://arxiv.org/abs/1710.10304.

[B44] Ribera J. He F. Chen Y. Habib A. F. Delp E. J. (2018) Estimating phenotypic traits from UAV based RGB imagery. Available at: http://arxiv.org/abs/1807.00498.

[B45] Rodionova M. V. Poudyal R. S. Tiwari I. Voloshin R. A. Zharmukhamedov S. K. Nam H. G.. (2017). Biofuel production: Challenges and opportunities. Int. J. Hydrogen Energy 42, 8450–8461. doi: 10.1016/j.ijhydene.2016.11.125

[B46] Shook J. Gangopadhyay T. Wu L. Ganapathysubramanian B. Sarkar S. Singh A. K. (2021). Crop yield prediction integrating genotype and weather variables using deep learning. PloS One 16 (6), e0252402. doi: 10.1371/journal.pone.0252402 34138872 PMC8211294

[B47] Sun S. Li C. Paterson A. H. Jiang Y. Xu R. Robertson J. S.. (2018). In-field high throughput phenotyping and cotton plant growth analysis using LiDAR. Front. Plant Sci. 9. doi: 10.3389/fpls.2018.00016 PMC5786533 29403522

[B48] Sundermeyer M. Schlüter R. Ney H. (2012) LSTM neural networks for language modeling. Available at: http://www.isca-speech.org/archive.

[B49] Tan C. Sun F. Kong T. Zhang W. Yang C. Liu C. (2018). “A survey on deep transfer learning,” in Artificial Neural Networks and Machine Learning–ICANN 2018. 11141, 270–279. doi: 10.1007/978-3-030-01424-7_27

[B50] Ullah A. Ahmad J. Muhammad K. Sajjad M. Baik S. W. (2017). “Action recognition in video sequences using deep bi-directional LSTM with CNN features,” in IEEE Access, 6, 1155–1166. doi: 10.1109/ACCESS.2017.2778011

[B51] van der Weijde T. Alvim Kamei C. L. Torres A. F. Vermerris W. Dolstra O. Visser R. G. F.. (2013). The potential of C4 grasses for cellulosic biofuel production. Front. Plant Sci. 4. doi: 10.3389/fpls.2013.00107 PMC3642498 23653628

[B52] Wang T. Crawford M. M. (2021). “Multi-year sorghum biomass prediction with UAV-based remote sensing data,” in in 2021 IEEE International Geoscience and Remote Sensing Symposium IGARSS. 4312–4315. doi: 10.1109/igarss47720.2021.9554313

[B53] Wang Y. H. Su W. H. (2022). Convolutional neural networks in computer vision for grain crop phenotyping: A review. Agronomy 12 (11), 2659. doi: 10.3390/agronomy12112659

[B54] Wang A. X. Tran C. Desai N. Lobell D. Ermon S. (2018). “Deep transfer learning for crop yield prediction with remote sensing data,” in Proceedings of the 1st ACM SIGCAS Conference on Computing and Sustainable Societies. 50, 1–5. doi: 10.1145/3209811.3212707

[B55] Wang Y. Yao Q. Kwok J. T. Ni L. M. (2020). Generalizing from a few examples: A survey on few-shot learning. ACM Comput. Surv. 53 (3), 1–34. doi: 10.1145/3386252

[B56] Xie Y. Huang J. (2021). Integration of a crop growth model and deep learning methods to improve satellite-based yield estimation of winter wheat in henan province, china. Remote Sens (Basel) 13, 4372. doi: 10.3390/rs13214372

[B57] Xie C. Yang C. (2020). A review on plant high-throughput phenotyping traits using UAV-based sensors. Comput. Electron Agric. 178, 105731. doi: 10.1016/j.compag.2020.105731

[B58] Yang K. W. Chapman S. Carpenter N. Hammer G. McLean G. Zheng B.. (2021). Integrating crop growth models with remote sensing for predicting biomass yield of sorghum. In Silico Plants 3 (1), diab001. doi: 10.1093/insilicoplants/diab001

[B59] Yang J. Guo X. Li Y. Marinello F. Ercisli S. Zhang Z. (2022). A survey of few-shot learning in smart agriculture: developments, applications, and challenges. Plant Methods 18 (1), 1–12. doi: 10.1186/s13007-022-00866-2 35248105 PMC8897954

[B60] Yang G. Liu J. Zhao C. Li Z. Huang Y. Yu H.. (2017). Unmanned aerial vehicle remote sensing for field-based crop phenotyping: Current status and perspectives. Front. Plant Sci. 8, 1111. doi: 10.3389/fpls.2017.01111 28713402 PMC5492853

[B61] Yang S. Zheng L. He P. Wu T. Sun S. Wang M. (2021). High-throughput soybean seeds phenotyping with convolutional neural networks and transfer learning. Plant Methods 17 (1), 50. doi: 10.1186/s13007-021-00749-y 33952294 PMC8097802

[B62] Yosinski J. Clune J. Bengio Y. Lipson H. (2014) How transferable are features in deep neural networks? Available at: http://arxiv.org/abs/1411.1792.

[B63] Zheng B. Dreccer F. Chapman S. Wang E. Chenu K. (2019). “Modelling the dynamic of canopy development in APSIM wheat,” in Agronomy Australia Conference, Wagga Wagga, Australia. 1–4.

[B64] Zhu X. X. Tuia D. Mou L. Xia G. S. Zhang L. Xu F.. (2017). “Deep learning in remote sensing: A comprehensive review and list of resources,” in IEEE Geosci Remote Sens Mag, 5. doi: 10.1109/MGRS.2017.2762307

[B65] Zimek A. Schubert E. Kriegel H. P. (2012). A survey on unsupervised outlier detection in high-dimensional numerical data. Stat. Anal. Data Min. 5, 363–387. doi: 10.1002/sam.11161

[B66] Zoph B. Yuret D. May J. Knight K. (2016) Transfer learning for low-resource neural machine translation. Available at: http://arxiv.org/abs/1604.02201.

