# Supplementary material for: A novel transfer learning framework for sorghum biomass prediction using UAV-based remote sensing data and genetic markers
Source: Front Plant Sci. 2023 Apr 11;14:1138479. doi: 10.3389/fpls.2023.1138479 (PMC10126475; doi:10.3389/fpls.2023.1138479)
Supplement: Supplementary file 1 [file DataSheet_1.docx]

Supplementary Table 1. The list of original hyperspectral features (Masjedi, 2020).

|  | Hyperspectral features |
| --- | --- |
| 1 | First derivative (FDR) slope |
| 2 | Minimum of FDR |
| 3 | Integration of FDR between 670 nm and 780 nm |
| 4 | Integration of FDR between 910 nm and 1000 nm |
| 5 | Second derivative (SDR) slope |
| 6 | Integration of SDR |
| 7 | Integration of blue (400 nm – 500 nm) |
| 8 | Integration of green (500 nm – 600 nm) |
| 9 | Integration of red (600 nm – 685 nm) |
| 10 | Integration of red edge (685 nm – 745 nm) |
| 11 | Integration of NIR1 (770 nm – 910 nm) |
| 12 | Integration of NIR2 (910 nm – 1000 nm) |
| 13 | NDVI |
| 14 | NDCI |
| 15 | Carte1 |
| 16 | SR800,680 |
| 17 | SR675,700 |
| 18 | SR700,670 |
| 19 | OSAVI |
| 20 | MCARI |
| 21 | REP |
| 22 | PRI |

Supplementary Table 2. The list of original LiDAR features (Masjedi, 2020).

|  | LiDAR features |
| --- | --- |
| 1 | 30 percentile height |
| 2 | 50 percentile height |
| 3 | 70 percentile height |
| 4 | 90 percentile height |
| 5 | 95 percentile height |
| 6 | 100 percentile height |
| 7 | Standard deviation of points height |
| 8 | Quadratic mean of points height |
| 9 | Skewness of points height |
| 10 | Kurtosis of points height |
| 11 | Volume |
| 12 | LiDAR canopy cover at 5 percentile height |
| 13 | LiDAR canopy cover at 5 percentile height |
| 14 | LiDAR canopy cover at 10 percentile height |
| 15 | LiDAR canopy cover at 20 percentile height |
| 16 | LiDAR canopy cover at 30 percentile height |
| 17 | LiDAR canopy cover at 40 percentile height |
| 18 | LiDAR canopy cover at 50 percentile height |
| 19 | LiDAR canopy cover at 75 percentile height |
