# Supplementary figures and images for: A novel transfer learning framework for sorghum biomass prediction using UAV-based remote sensing data and genetic markers
Source: Front Plant Sci. 2023 Apr 11;14:1138479. doi: 10.3389/fpls.2023.1138479 (PMC10126475; doi:10.3389/fpls.2023.1138479)

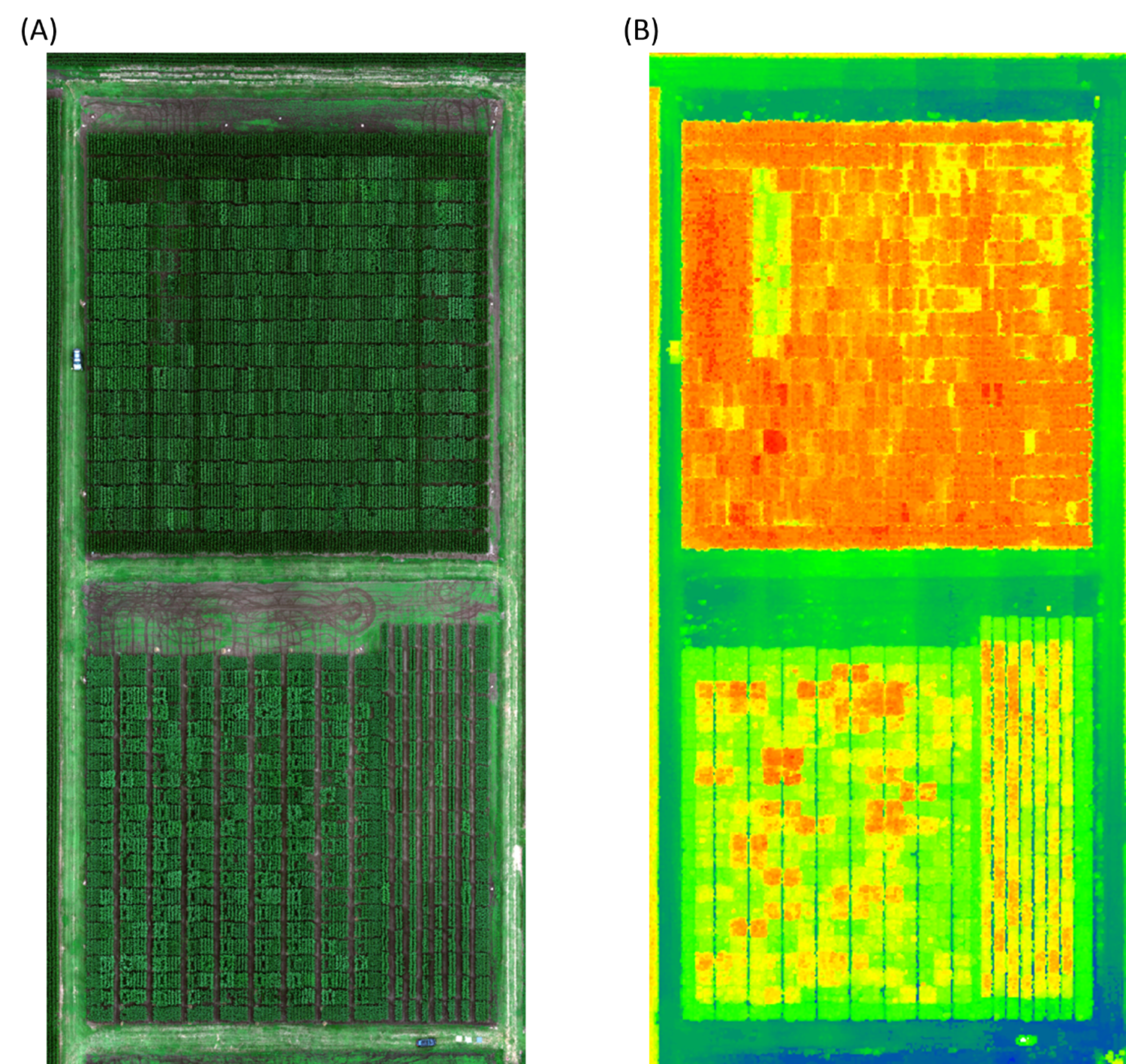

Supplement: Supplementary Figure 1 — An example of (A) hyperspectral orthomosaic, and (B) reconstructed LiDAR point cloud over field 54. [file Image_1.tif]

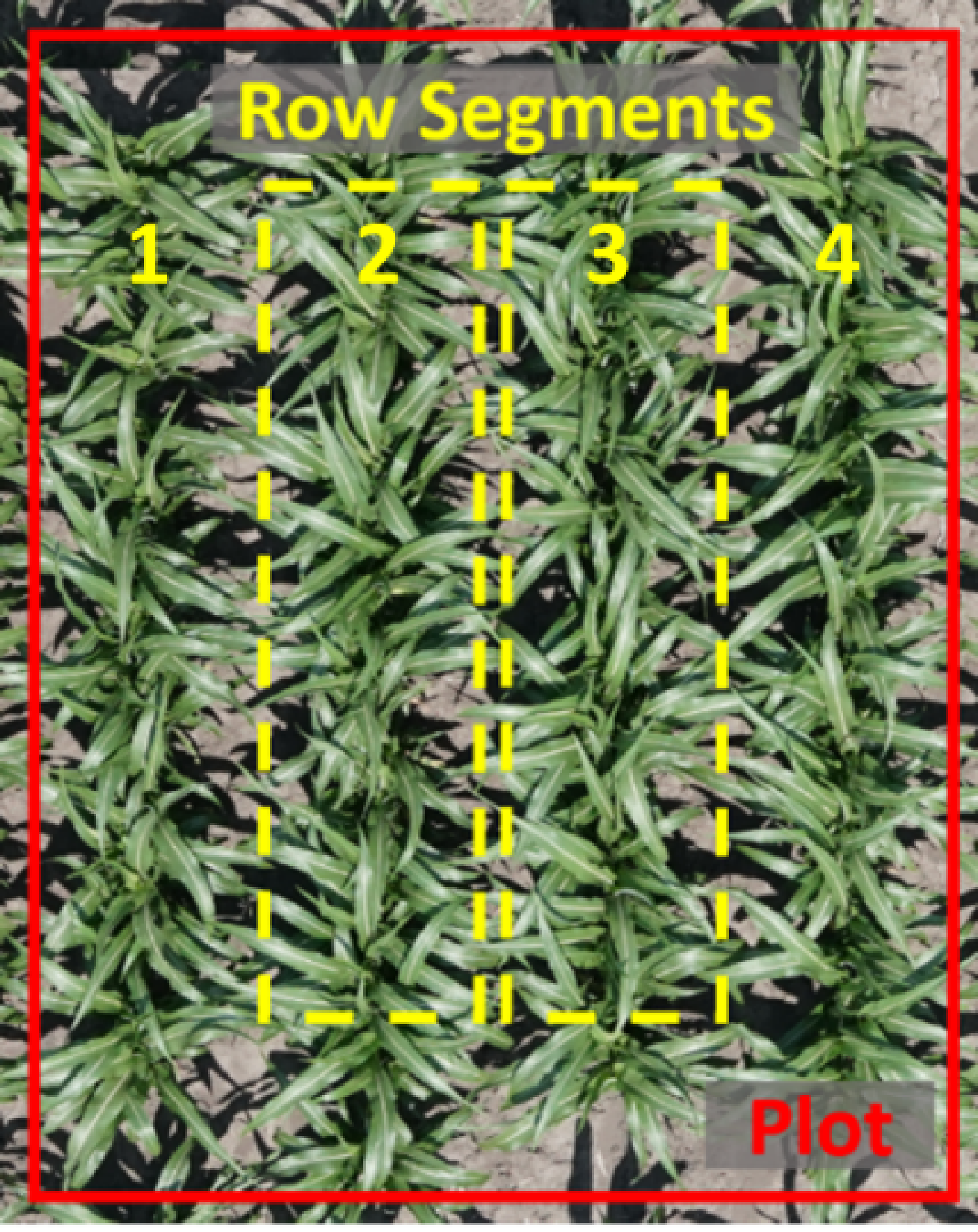

Supplement: Supplementary Figure 2 — Example 4-row plot from the Tc panel in 2020; Features extracted from areas within the yellow boxes. [file Image_2.tif]

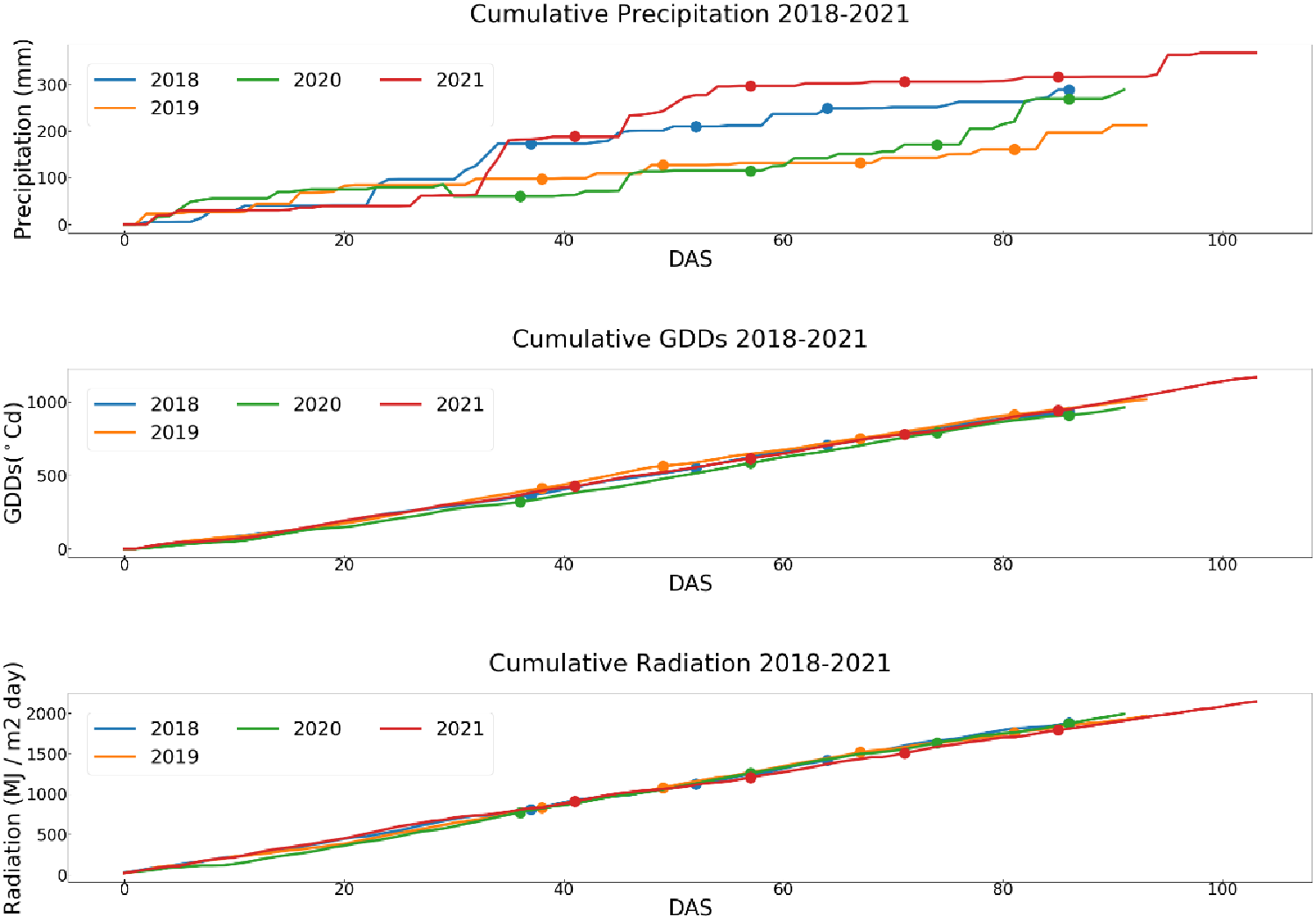

Supplement: Supplementary Figure 3 — Cumulative environmental features during 2018-2021 growing season: (A) cumulative precipitation, (B) cumulative GDDs, and (C) cumulative radiation. [file Image_3.tif]

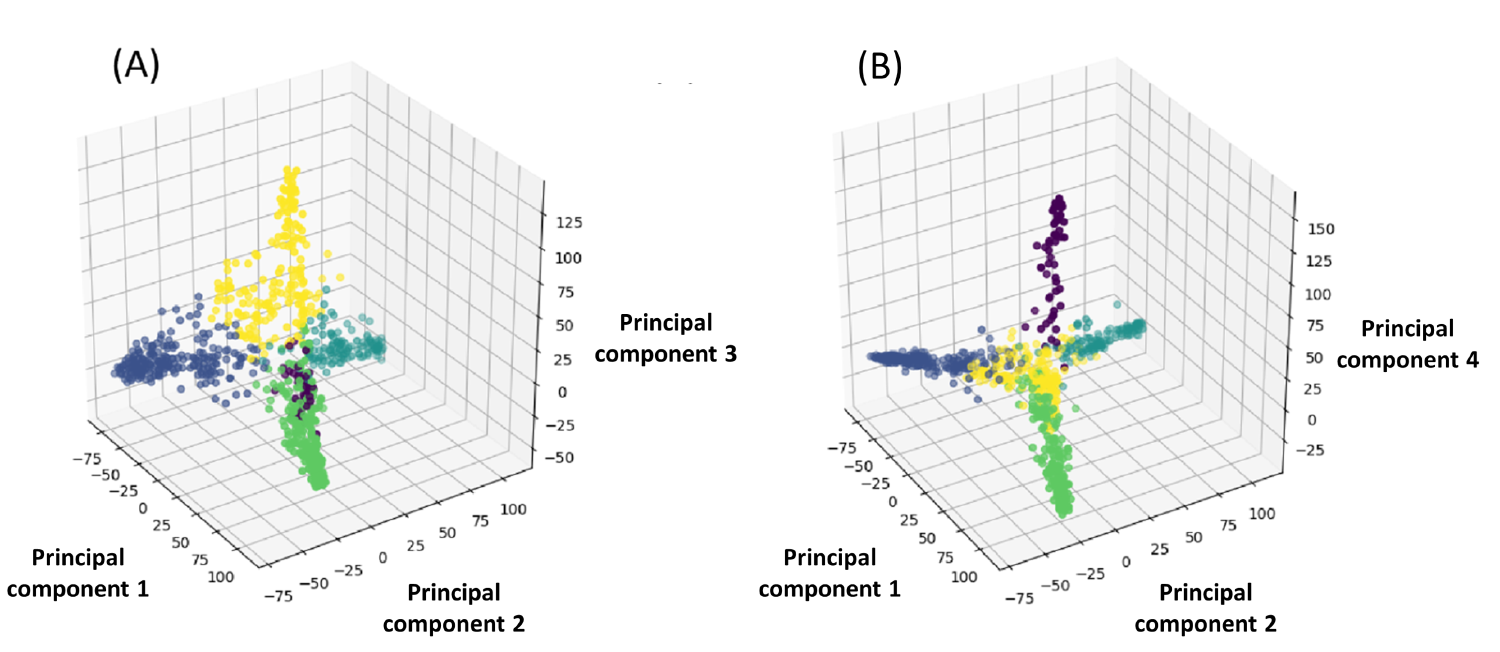

Supplement: Supplementary Figure 4 — 3D plots of the five genotype clusters in different colors: (A) plot showing the three principal components (PCs) PC1, PC2, and PC3, and (B) plot showing PC1, PC2, and PC4. [file Image_4.tif]

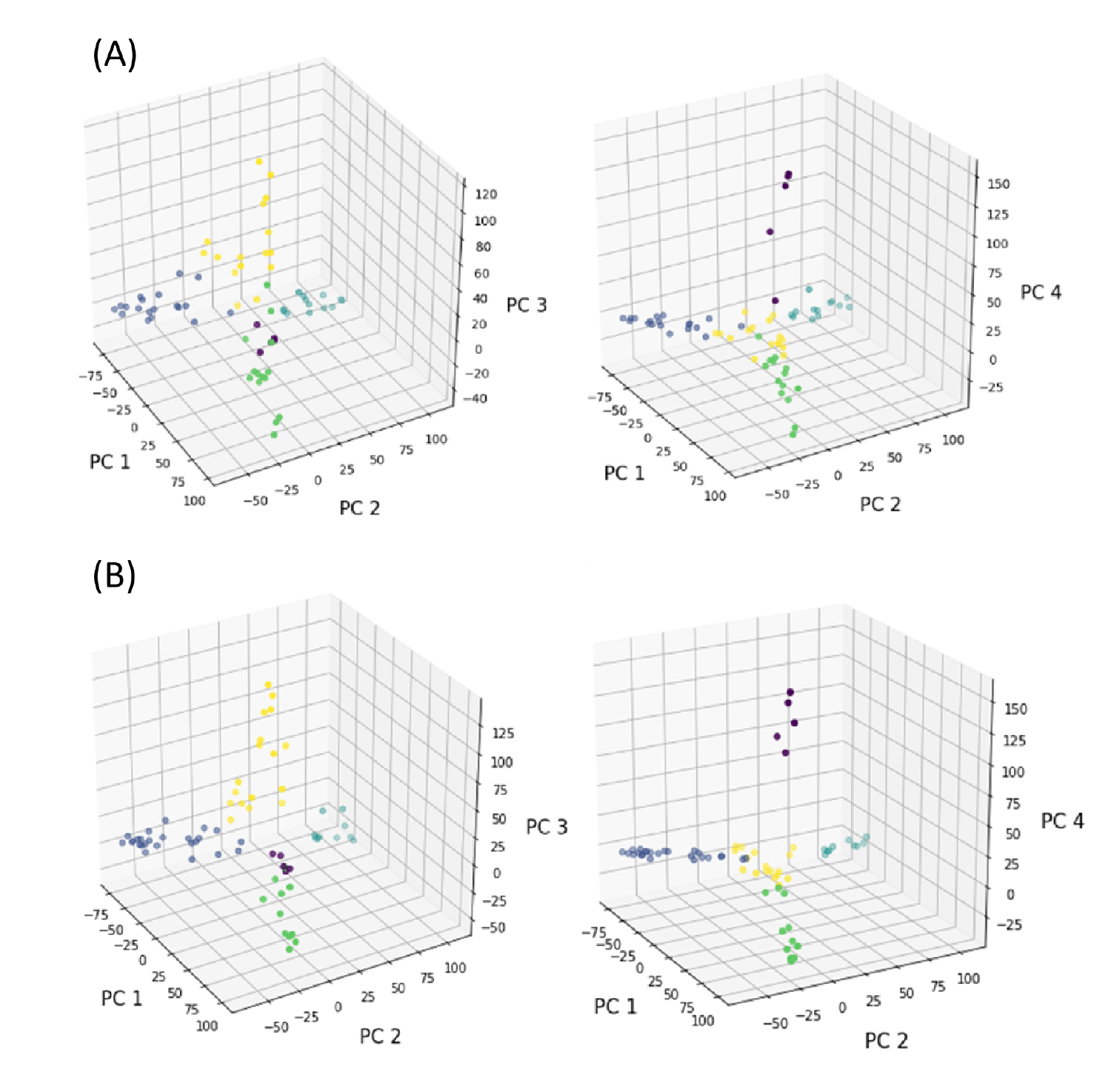

Supplement: Supplementary Figure 5 — Genotype clusters of the training samples selected by two transfer learning strategies, shown in 3D plots with different combinations of principal components (PCs), (A) shows the 67 hybrids selected by R19cal-G19 and the distribution of those hybrids in all hybrid lines; (B) shows the 68 hybrids selected by R19cal-P19 and the distribution of those hybrids in all hybrid lines. [file Image_5.tif]

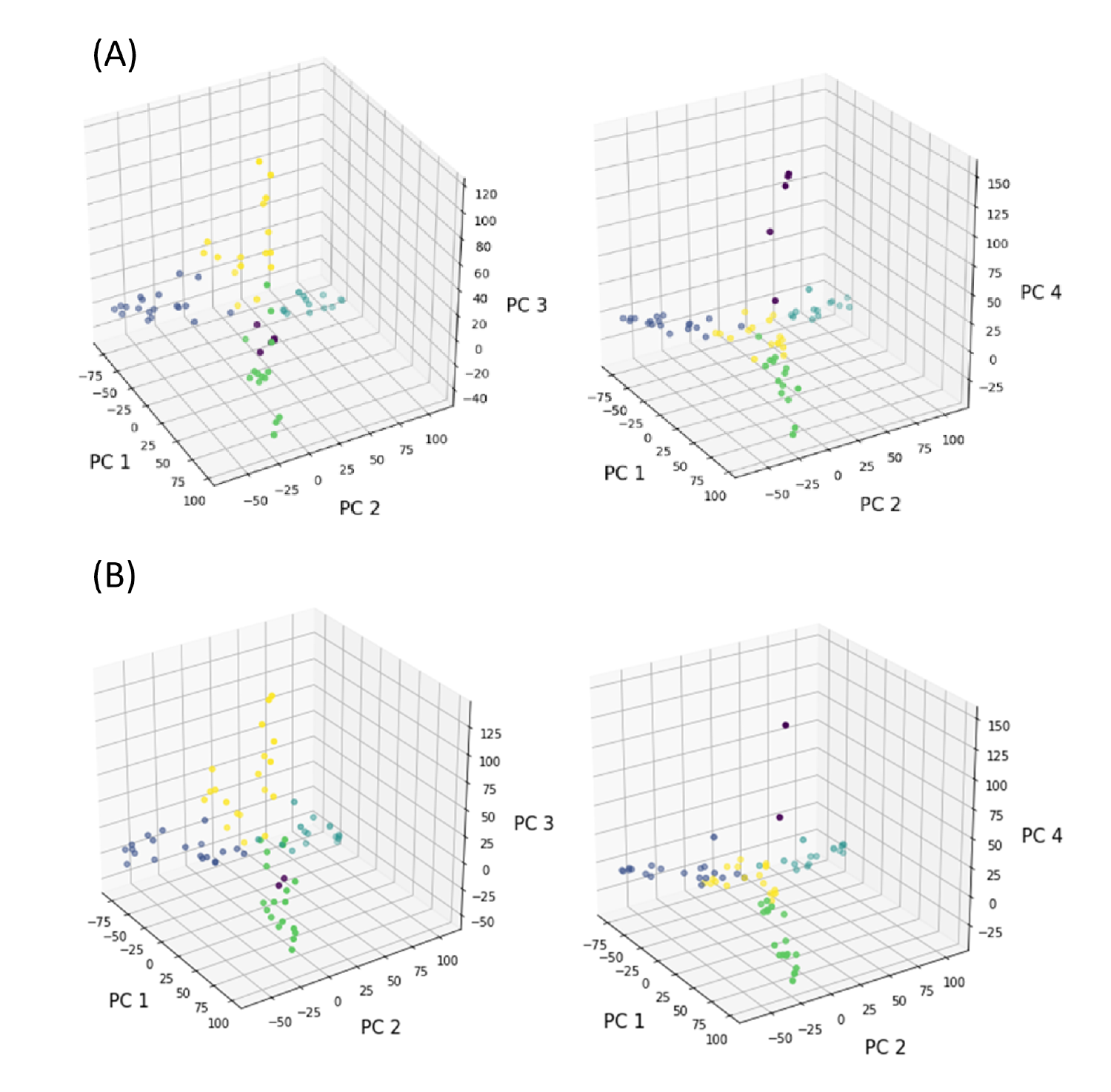

Supplement: Supplementary Figure 6 — Genotype clusters of the training samples selected by two transfer learning strategies, shown in 3D plots with different combinations of principal components (PCs), (A) shows the 72 hybrids selected by R20cal-G20 and the distribution of those hybrids in all hybrid lines; (B) shows the 77 hybrids selected by R20cal-P20 and the distribution of those hybrids in all hybrid lines. [file Image_6.tif]

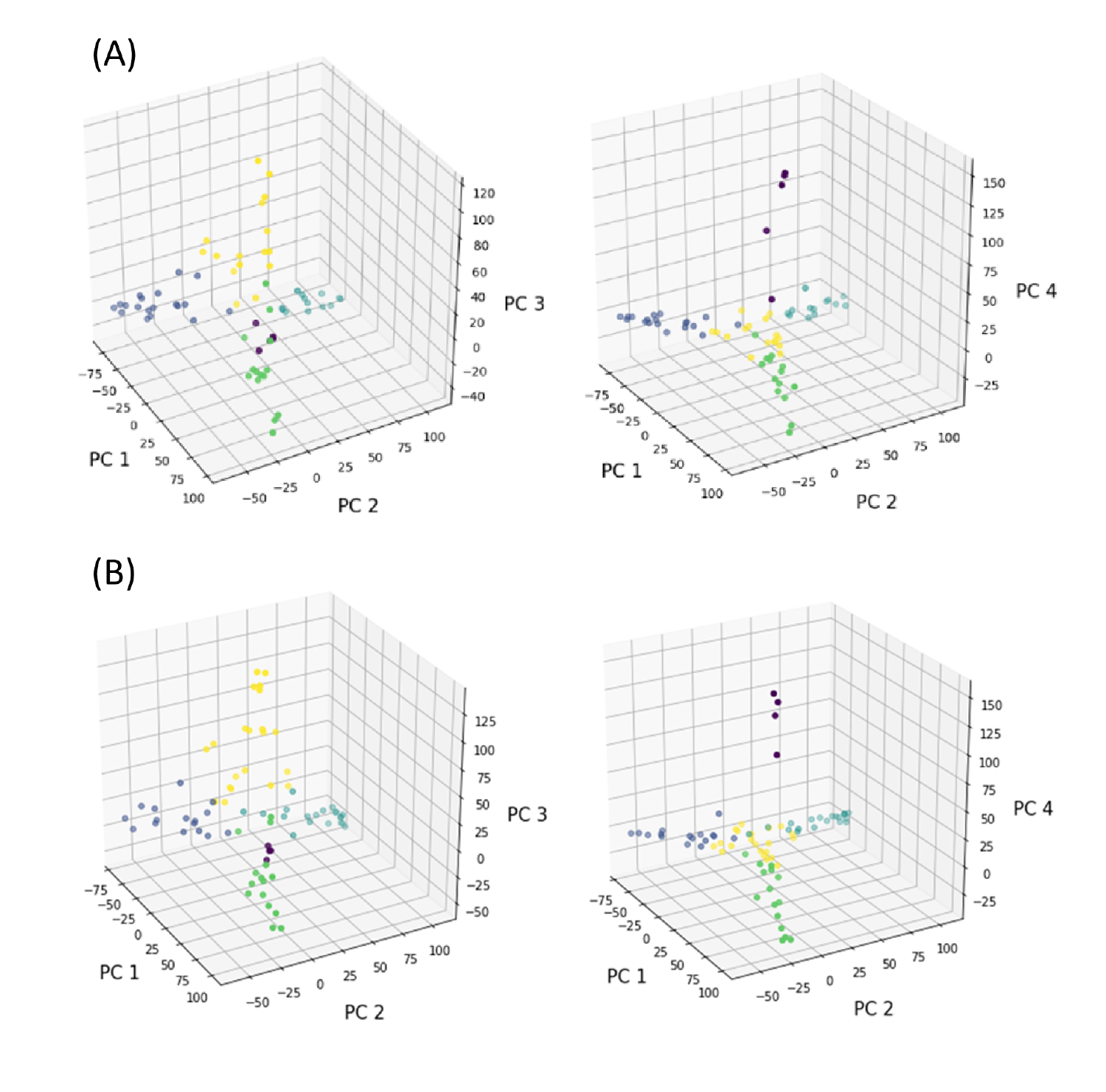

Supplement: Supplementary Figure 7 — Genotype clusters of the training samples selected by two transfer learning strategies, shown in 3D plots with different combinations of principal components (PCs), (A) shows the 67 hybrids selected by R21cal-G18 and the distribution of those hybrids in all hybrid lines; (B) shows the 76 hybrids selected by R21cal-P18 and the distribution of those hybrids in all hybrid lines. [file Image_7.tif]
